# Range‐wide population genomics of the spongy moth, *Lymantria dispar* (Erebidae): Implications for biosurveillance, subspecies classification and phylogeography of a destructive moth

**DOI:** 10.1111/eva.13522

**Published:** 2023-01-13

**Authors:** Sandrine Picq, Yunke Wu, Vyacheslav V. Martemyanov, Esther Pouliot, Scott E. Pfister, Richard Hamelin, Michel Cusson

**Affiliations:** ^1^ Laurentian Forestry Centre Natural Resources Canada Quebec Quebec City Canada; ^2^ United States Department of Agriculture, APHIS, PPQ, Science and Technology Forest Pest Methods Laboratory Massachusetts Buzzards Bay USA; ^3^ Department of Ecology and Evolutionary Biology Cornell University New York Ithaca USA; ^4^ Institute of Systematics and Ecology of Animals SB RAS Novosibirsk Russia; ^5^ Biological Institute National Research Tomsk State University Tomsk Russia; ^6^ Department of Forest and Conservation Sciences The University of British Columbia British Columbia Vancouver Canada; ^7^ Département de biochimie, de microbiologie et de bio‐informatique Université Laval Quebec Quebec City Canada

**Keywords:** genetic cline, genotyping‐by‐sequencing SNPs, gypsy moth, invasive species, population assignment, sample size

## Abstract

The spongy moth, *Lymantria dispar*, is an irruptive forest pest native to Eurasia where its range extends from coast to coast and overspills into northern Africa. Accidentally introduced from Europe in Massachusetts in 1868–1869, it is now established in North America where it is considered a highly destructive invasive pest. A fine‐scale characterization of its population genetic structure would facilitate identification of source populations for specimens intercepted during ship inspections in North America and would enable mapping of introduction pathways to help prevent future incursions into novel environments. In addition, detailed knowledge of *L. dispar*'s global population structure would provide new insight into the adequacy of its current subspecies classification system and its phylogeographic history. To address these issues, we generated >2000 genotyping‐by‐sequencing‐derived SNPs from 1445 contemporary specimens sampled at 65 locations in 25 countries/3 continents. Using multiple analytical approaches, we identified eight subpopulations that could be further partitioned into 28 groups, achieving unprecedented resolution for this species' population structure. Although reconciliation between these groupings and the three currently recognized subspecies proved to be challenging, our genetic data confirmed circumscription of the *japonica* subspecies to Japan. However, the genetic cline observed across continental Eurasia, from *L. dispar asiatica* in East Asia to *L. d. dispar* in Western Europe, points to the absence of a sharp geographical boundary (e.g., the Ural Mountains) between these two subspecies, as suggested earlier. Importantly, moths from North America and the Caucasus/Middle East displayed high enough genetic distances from other populations to warrant their consideration as separate subspecies of *L. dispar*. Finally, in contrast with earlier mtDNA‐based investigations that identified the Caucasus as *L. dispar*'s place of origin, our analyses suggest continental East Asia as its evolutionary cradle, from where it spread to Central Asia and Europe, and to Japan through Korea.

## 1 INTRODUCTION

Genetic delimitation of distinct population units of a potentially invasive species plays a key role in assessing risk of establishment, identifying source populations of intercepted specimens or mapping introduction pathways. However, the often complex evolutionary history of invasive species, where human‐aided movement can be a confounding factor, makes it difficult to delineate population units using only a small number of mitochondrial or nuclear makers. Recently, the use of genome‐wide markers has helped overcome some of these problems, making it possible to successfully resolve regional‐ and local‐scale population genetic structure of important invasive species, such as the Formosan subterranean termite, *Coptotermes formosanus* (Blumenfeld et al., 2021), or the cabbage butterfly, *Pieris rapae* (Ryan et al., 2019). The spongy moth (previously known as the gypsy moth), *Lymantria dispar*, is recognized as one of the most threatening invasive species in the world (Lowe et al., 2000), and many studies have begun assessing genetic differentiation of its populations, but typically using a limited number of molecular markers (e.g. Keena et al., 2008; Wu et al., 2015; Zahiri et al., 2019; Zhao et al., 2019). A high‐resolution characterisation of this insect's spatial genetic structure over its entire geographic range, using thousands of single nucleotide polymorphism (SNP) markers, would thus constitute an invaluable resource in support of efforts aimed at preventing the establishment and spread of this pest into novel environments.

In North America, the spongy moth is a highly destructive pest of broad‐leaf trees, with an economic cost estimated at US$3.2 billion per year (Bradshaw et al., 2016). Subsequent to its accidental introduction from Europe to Massachusetts, USA, in 1868–1869 (Forbush & Fernald, 1896), the spongy moth spread to neighboring US states and Canadian provinces, resulting in a distribution that currently extends from Virginia to southern Quebec, along the east coast, and to Minnesota via southern Ontario inland (for historical details on the spread, see figure 2.4 and figure 1 in Liebhold et al., 2007, 2021, respectively). Its native range is vast, with a nearly coast‐to‐coast presence in Eurasia, and populations established in northern Africa (Fuester et al., 2014; Pogue & Schaefer, 2007). It has long been recognized that in different parts of this extensive range there exist *L. dispar* populations with distinct biological attributes suggestive of underlying genetic differences. For instance, the early work of Goldschmidt (1934, 1940) pointed to the existence of a “chain of geographic races” across the spongy moth's range, which led to his recognition of at least seven subspecies. While classification of spongy moth subspecies has been and remains a controversial issue, with many proposed revisions published since the work of Goldschmidt (e.g. Inoue et al., 2019; Kishida, 2011; Schintlmeister, 2004; Zahiri et al., 2019), the revision of Pogue and Schaefer (2007) has gained some degree of authority. These authors recognized three subspecies of *L. dispar*, namely (i) *L. dispar japonica*, found on the main islands of the Japanese archipelago, including the western portion of Hokkaido, (ii) *L. dispar asiatica*, present in continental Asia, from the Ural Mountains to the Russian Far East, with a high prevalence in eastern China and South Korea, and (iii) *L. dispar dispar*, observed in Europe, west of the Ural Mountains, north Africa and eastern North America. Along with two closely related *Lymantria* species found in eastern Hokkaido (*L. umbrosa*) and the Japanese Ryukyu Islands (*L. albescens*; see Djoumad et al., 2020, for a recent taxonomic revision), the two Asian subspecies of *L. dispar* are now collectively referred to as Asian spongy moths, largely on the basis of a shared biological trait believed to confer greater invasive propensity, that is female flight capability. In comparison, females of *L. dispar dispar*, now referred to as European spongy moths, are flight‐incapable. This *L. dispar* classification is currently used as a guide by North American plant protection regulatory agencies in their efforts to prevent accidental introductions of Asian spongy moth now considered to pose a greater invasive threat than the European subspecies introduced 150 years ago.

Although the above subspecies classification has provided a reasonable framework for regulatory operations and has led to the development of various molecular tools that help identify *L. dispar* subspecies in egg masses intercepted on marine vessels entering North American ports (e.g., Stewart et al., 2016), it should be recognized that the underlying genetic makeup of these populations could be more complicated than previously thought. The three‐subspecies scheme appears to hold up in mtDNA‐based phylogenetic analyses where central Asian and Caucasian populations are not taken into account (Wu et al., 2015) or in nuclear marker‐based analyses focusing on a subset of geographic populations (Picq et al., 2018). However, studies that examined populations of the spongy moth across its entire range, using either microsatellite markers or a combination of 60 nuclear and mitochondrial loci, recovered 4 or 5 distinct major population clusters, with North American spongy moths forming one of them (Wu et al., 2015, 2020). In addition, there appears to be a *dispar‐asiatica* cline within a region extending from Eastern Europe to central Asia, where nuclear markers show the existence of admixture between these two subspecies (Picq et al., 2018; Wu et al., 2015, 2020). Interestingly, spongy moths in this zone are most often genotyped as *L. dispar dispar*, using mitochondrial markers (Djoumad et al., 2017; Martemyanov et al., 2019; Zahiri et al., 2019; Zhao et al., 2019), an observation that suggests some degree of mito‐nuclear discordance in these populations. A second clinal zone, encompassing northeastern China, the Korean Peninsula, and the Russian Far East, has also been reported for the subspecies *asiatica* and *japonica* (Wu et al., 2015, 2020). Finally, recent studies have uncovered the existence of a very distinct mitochondrial genotype in spongy moth populations found in and around the Caucasus (Djoumad et al., 2017; Zhao et al., 2019), leading some authors to suggest that they form a distinct subspecies (Zahiri et al., 2019). However, a principal component analysis examining genome‐wide SNPs recently led to the conclusion that two specimens from this region could not be differentiated from European moths (Zhang et al., 2019). A thorough characterization of nuclear genome‐wide differentiation will provide a basis for assessing the adequacy of the current subspecies classification system and determining whether Caucasian populations form a differentiated group.

Beyond helping on subspecies delimitation, the genetic characterization of spongy moth populations can assist in tracking the invasive and evolutionary history of this Holarctic species. Indeed, molecular markers can assist in tracing the exact source of the moths that founded the North American population. With respect to the demographic history of the spongy moth, it has generally been assumed that the insects accidentally introduced in Massachusetts by French naturalist Leopold Trouvelot were from France, a claim that found some support in the early work of Harrison et al. (1983) and Harrison and ODell (1989) and more recently in Wu et al. (2015). Other analyses, however, reported that North American moths had mtDNA haplotypes closer to those of moths from Germany and Sardinia than to those from France (Bogdanowicz et al., 2000; Wu et al., 2015), although statistical support was low. In addition, population genetic analyses can help assess whether subsequent, undetected introductions of spongy moths from Europe or Asia have resulted in significant admixture within New World populations. For example, in the early 1990's, important outbreaks in the Russian Far East and in Germany (Prasher & Mastro, 1995; Savotikov et al., 1995; Wulf & Graser, 1996) were the source of introduction of egg masses via infested ships or military equipment repatriation. Furthermore, fine‐scale population genetic structure of the spongy moth can be used to identify the geographic sources of specimens intercepted in North American ports during ship inspections (Picq et al., 2018). Finally, the genetic differentiation pattern can also lay the foundation for a phylogeographic assessment of competing hypotheses as to where the species *L. dispar* actually arose (see Bogdanowicz et al., 2000; Goldschmidt, 1934; Harrison et al., 1983; Wu et al., 2015; Zahiri et al., 2019; Zhang et al., 2019).

The present study reports on an in‐depth characterization of the spongy moth's population genomic structure, developed from a large collection of contemporary specimens (2013–2018) sampled across *L. dispar*'s entire geographic range, and based on >2000 genome‐wide SNPs generated through a genotyping‐by‐sequencing (GBS) approach. Many earlier studies have assessed population differentiation in this species, using different types of molecular markers and analytical approaches, as well as a varying assortments of focal populations, sample sizes and specimen ages (e.g. Bogdanowicz et al., 1997; Chen et al., 2016; Harrison et al., 1983; Harrison & ODell, 1989; Kang et al., 2017; Keena et al., 2008; Lacković et al., 2015, 2018; Martemyanov et al., 2019; Picq et al., 2018; Wu et al., 2015, 2020; Xu et al., 2019; Zhao et al., 2019). To our knowledge, however, the present work represents the most extensive and thorough assessment conducted to date. Here, we used several analytical approaches to characterize population differentiation in the spongy moth and to address some of the issues raised above, namely: (i) determine whether the current subspecies classification is supported by genome‐wide data, (ii) assess whether the Caucasian populations form a genomically differentiated group, (iii) identify the source of the moths that founded the North American population, and finally (iv) retrace the evolutionary history of *L. dispar* in its native range.

## 2 MATERIALS AND METHODS

### 2.1 Moth sampling

**FIGURE 1:**
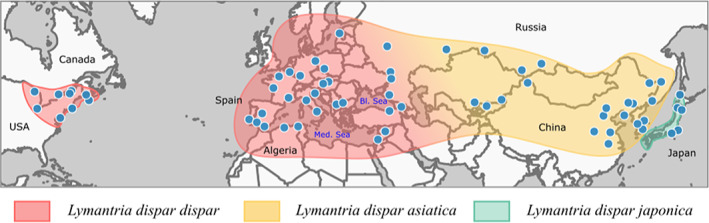
*Lymantria dispar* sampling locations used in this study. Each dot represents a sampling site within the species' geographic range. Approximate distributions of the three *L. dispar* subspecies recognized by Pogue and Schaefer (2007) are represented by distinct shading colors. Range boundary in the northern portion of Asia is not yet well known; the border shown here follows the southern limit of the taiga.

The bulk of spongy moth specimens were collected during the summers of 2017 and 2018 using milk‐carton type pheromone‐baited traps. Each trap was equipped with a Vaportape® insecticidal strip (Hercon Environmental) and baited with synthetic female spongy moth sex pheromone (“disparlure”: cis‐7,8‐epoxy‐2‐methyloctadecane; string dispenser, PHEROCON® Trécé Inc., Distributions Solida Inc.). For moth collection, we relied on a network of colleagues and contacts at different locations across the spongy moth's range (Table S1). Captured specimens were placed in large glassine envelopes, labeled with the location name and collection date, and stored frozen until shipment to our laboratory. Additional samples (whole moths or parts thereof) from regions not fully covered by our network of traps were provided by colleagues who had collected them in the context of independent studies; with the exception of a few samples, collection dates for these were recent (overall, 93% of the moths used were collected between 2013 and 2018, and most specimens were males [only 0.8% of females; Table S2]). Once in our laboratory, moths were placed individually in glassine envelopes and stored at −20°C until processed. Among the approximately 18,000 moths that were collected, we selected 1445 individuals captured at 65 sampling sites in 25 different countries. This subsampling scheme was designed to provide optimal coverage of the full geographic range of *L. dispar* (Table 1; Figure 1; Figure S1). For some regions where trap catches were low, we pooled moths from sampling locations considered to be near one another to increase sample size (Table 1; Table S2).

**TABLE 1:** Information on sampling locations

No.	Country	Site code	Site—region	Latitude	Longitude	Date of collection	Number of moths^a^
North America							
1	Canada	CA07	Denwood—Ontario	46.51	−84.26	2017	30 (30)
2	Canada	CA04	Plaisance—Québec	45.60	−75.05	2017	30 (30)
3	Canada	CA03	Victoriaville—Québec	46.05	−71.95	2017	29 (30)
4	Canada	CA17	Saint Nicolas—Québec	46.68	−71.36	2017	27 (30)
5	Canada	CA13	Gagetown—New Brunswick	45.75	−66.19	2017	29 (30)
6	Canada	CA38	Kejimkujik—Nova Scotia	44.39	−65.20	2017	21 (30)
7	USA	US01^b^	Detroit—Michigan	42.27	−83.31	2017	22 (30)
8	USA	US02^b^	Boston—Massachusetts	42.31	−71.07	2016	29 (30)
9	USA	US04^b^	Philadelphia—New Jersey	39.83	−75.26	2016	21 (24)
Europe							
10	Portugal	PT01	Cascais—Lisbon	38.70	−9.42	2018	26 (30)
11	Spain	SP01	Mijares—Ávila	40.30	−4.84	2018	30 (30)
12	Spain	SP02^b^	El Castaño—Andalucia	–	–	1994	15 (20)
13	Spain	SP07^b^	Bancales de Pote—Andalucia	36.75	−4.04	2018	26 (30)
14	Algeria	DZ02	Chréa—Blida	36.43	2.89	2017	20 (20)
15	Algeria	DZ03	Seraidi—Annaba	36.75	7.76	2017	19 (20)
16	France	FR05	Pouzauges—Vendée	46.74	−0.89	2017	28 (30)
17	France	FR06	Labastide de Virac—Ardèche	44.35	4.41	2017	30 (30)
18	France	FR08	Le petit quévilly—Seine‐Maritime	49.43	1.06	2017	28 (30)
19	Belgium	BE01	Malonne—Namur	50.42	4.80	2017	25 (30)
20	Italy	IT01	Bientina—Tuscany	43.70	10.64	2017	28 (30)
21	Italy	IT04	Portici—Campania	40.81	14.35	2017	28 (30)
22	Germany	DE01	Eberswalde—Brandenburg	52.82	13.81	2017	17 (18)
23	Germany	DE03	Lampertheim—Hesse	49.70	8.53	2015	18 (20)
24	Slovenia	SI01	Dragonja—Slovene Littoral	45.45	13.66	2017	28 (30)
25	Austria	AT02	Siegendorf—Burgenland	47.76	16.56	2017	28 (30)
26	Slovakia	SK01	Cifare—Zvolen	48.21	18.40	2017	30 (30)
27	Poland	PL01	Wroclaw—Lower Silesian Voivodeship	51.15	16.95	2017	30 (30)
28	Kosovo	XR01	Priština—District of Priština	42.67	21.18	2017	10 (14)
29	Bulgaria	BG01	Levishte—Sofia	43.08	23.46	2017	27 (30)
30	Estonia	EE01	Kassiku—Saare County	58.28	22.27	2018	30 (30)
31	Russia	RU41	Orekhovo‐Zouïevo—Moscow Oblast	55.8	38.97	2017	16 (30)
32	Russia	RU05	Pavlovsk—Krasnodar Krai	50.45	40.12	2015	2 (12)
33	Russia	RU07	Leskhoz—Rostov Oblast	48.91	40.4	2015	7 (18)
34	Russia	RU38^b^	Caucasus	45.05	39.72	2018	18 (25)
35	Georgia	GE01^b^	Telavi—Kakheti	41.91	45.305	2010–2014	8 (9)
36	Turkey	TR01	Çilekli—Trabzon	40.96	39.77	2017	29 (30)
37	Israel	IS01	Golan Heights	–	–	2017	19 (20)
38	Syria	SY02	Unknown	–	–	2008	10 (10)
Central Asia							
39	Russia	RU24^b^	Oural	55.19	59.72	2018^c^	21 (28)
40	Russia	RU08^b^	Novossibirsk	54.97	73.09	2015	15 (19)
41	Russia	RU28^b^	Altay	50.84	86.16	2017^c^	9 (16)
42	Russia	RU01	Verhneusinsk—Krasnoyarsk Krai	52.24	93.03	2017	10 (10)
43	China	CN06	Altay—Xinjiang	47.97	88.20	2017	5 (5)
44	Kazakhstan	KZ01^b^	Turkistan Region	43.20	69.62	2015	5 (6)
45	Kazakhstan	KZ04^b^	Almaty Region	43.87	79.07	2015	11 (17)
46	Kyrgyzstan	KG01^b^	Chuy region	42.25	73.83	2015	4 (5)
East Asia							
47	China	CN01	Songshan—Beijing	40.50	115.85	2017	30 (30)
48	China	CN02	Zhanjiakou—Hebei	40.90	115.75	2017	30 (30)
49	China	CN07	Jinzhong—Shanxi	35.32	111.52	2017	5 (5)
50	China	CN08	Liu'an—Anhui province	31.74	116.52	2013	7 (10)
51	China	CN09	Tengzhou—Shandong province	35.08	117.15	2013	9 (10)
52	China	CN10	Liauyuan—Jilin province	42.88	125.15	2013	10 (10)
53	China	CN03	Wafangdian—Liaoning	39.79	122.07	2017	15 (15)
54	China	CN05	Tongliao—Inner Mongolia	43.10	123.48	2017	30 (30)
55	China	CN04	Hegang—Heilongjiang	47.91	130.87	2017	29 (30)
56	Russia	RU02^b^	Primorsky Krai	43.56	131.86	2018^c^	12 (16)
57	Russia	RU39	Khabarovsk—Khabarovsk Krai	48.20	135.00	2018	29 (30)
58	South Korea	KR02	Yeongju—Yeongnam	36.83	128.53	2017	25 (30)
59	South Korea	KR03	Donghae—Gangwon	37.50	129.08	2009	7 (10)
60	South Korea	KR04	Incheon—Incheon	37.46	126.65	2009	7 (10)
61	Japan	JP02^b^	Tokyo—Honshu	–	–	2015	18 (20)
62	Japan	JP03^b^	Misawa air force base—Honshu	40.86	141.38	2018^c^	16 (38)
63	Japan	JP04	Sapporo—Hokkaido	45.10	141.59	1992	8 (10)
64	Japan	JP07^b^	Chiba—Honshu	35.56	140.11	2007^c^	7 (15)
65	Japan	JP08	Hakodata—Hokkaido	41.78	140.71	2007	7 (10)

^a^ No. of individuals analyzed (No. of individuals sampled).

^b^ Sites for which moths from several nearby locations were pooled. Geographic coordinates are the averages of latitudes and longitudes of the different sampling locations.

^c^ Sites for which moths from two successive years of sampling were pooled.

### 2.2 DNA extraction and sequencing

For DNA extraction, we sampled one antenna and three legs from each moth. These were frozen in liquid nitrogen and ground using a Retsch MM 200 mixer mill (Retsch Technology). Then, DNA was extracted with the DNeasy 96 Blood & Tissue Kit (Qiagen) following the manufacturer's instructions, with the exception of an additional RNase A treatment before the addition of buffer AL/ethanol (4 μl of 100 mg/ml Rnase A; 5 min digestion at room temperature). DNA concentration and purity of the extracts were assessed using a NanoDrop 8000 spectrophotometer (Thermo Scientific). Samples were diluted to 10 ng/μl prior to library construction. Libraries were prepared based on a genotyping‐by‐sequencing (GBS) protocol using the restriction enzymes *Pst*I and *Msp*I (Poland et al., 2012). Individuals were barcoded with unique sequences and pooled in multiplexes of 96 individuals per library. Moths from the same sampling site were randomized in the different libraries to reduce the chances of artifactual library effects being interpreted as a biological pattern. Library preparation and sequencing on Ion Torrent Proton P1v2 chips were carried out at the Genomic Analysis Platform of Université Laval, Quebec City, Canada (for a detailed description of the method, see Abed et al., 2019).

### 2.3 SNP genotyping

Read quality was first evaluated using the FastQC software, a quality‐control tool for raw sequence data generated by high‐throughput sequencing pipelines (Andrews, 2016). Then, SNPs were discovered and called using Tassel 3.0 GBS (Glaubitz et al., 2014), with physical alignment to the *L. dispar asiatica* reference genome (lda.genome.v0.3.masked.fasta, Hebert et al., 2019), using the Burrows‐Wheeler Aligner (BWA) (Li & Durbin, 2009). At the time of conducting the present work, the *L. dispar asiatica* reference genome was considered the most complete *L. dispar* genome assembly available. The Tassel 3.0 GBS tool was particularly suited to our dataset as it is designed for processing data with low sequencing depth (0.5 to 3×) produced over a large number of markers in a large sample of individuals. Briefly, the pipeline retains only reads displaying a perfect match to a barcode and having no missing data in the following 64 bp. Identical reads are then clustered into tags, and only tags sustained by a minimum number *c* ≥ 5 (user‐specified) of reads across all samples are kept for further processing. Then, the tags are aligned to the reference genome using the external software BWA and the built‐in *aln* algorithm (default values). During this step, tags with multiple or unknown physical genomic positions are discarded. Tassel 3.0 GBS performs quantitative SNP calling, i.e., the minimum number of reads of the minor allele needed to call a heterozygote genotype is defined by both the sequencing error rate (default value 0.01) and the sum of the number of reads for the two alleles.

### 2.4 SNP filtering

Missing data filtering was initiated with low cut‐off values (genotype call rates ≥50% and individuals with ≤90% of missing data) that were then iteratively and alternately increased to obtain a final data set with a genotype call rate ≥80% comprising individuals with ≤25% of missing data. This gradual filtering is known to result in the retention of more loci and individuals than a one‐step hard cut‐off (O'Leary et al., 2018). Then, SNPs with a minor allele frequency (MAF) <0.01 across populations were removed. This filtering step excludes potential sequencing errors and uninformative polymorphism that may bias analyses using *F*_ST_ calculations or other divergence metrics (Roesti et al., 2012). Remaining sequencing and genotyping errors were discarded by removing SNPs with heterozygosity >0.5 (Hohenlohe et al., 2011) and in Hardy–Weinberg disequilibrium (*p*‐value <0.001) in at least 6 populations (Chen et al., 2017; Graffelman et al., 2017; Teo et al., 2007). Deviation from Hardy–Weinberg Equilibrium was tested using an exact test (Wigginton et al., 2005) performed on each of the 28 populations containing ≥25 individuals. To reduce genetic linkage among SNPs in our data set (i) we used only the first SNP in each tag and then (ii) we used the SNP most often genotyped in pairs of SNPs in different tags showing an *r* ^2^ value ≥0.8 in at least 6 populations. Indeed, SNPs physically linked can introduce bias in different classical analyses requiring independence of loci such as SNP outlier detection and certain population clustering method (Helyar et al., 2011). The different filtering procedures were carried out using VCFtools v0.1.16 (Danecek et al., 2011), and the resulting VCF file was converted to file formats suitable for each subsequent analysis using *PGDSpider v2.1.1.5* (Lischer & Excoffier, 2012).

### 2.5 Detection of loci under putative selection

Genetic variation at neutral loci results from the combined action of mutations, drift and gene flow. For adaptive loci, variation is also influenced by selection. Thus, neutral and adaptive loci may reveal different population structures depending on spatial patterns of genetic drift, gene flow and selection (Funk et al., 2012; Luikart et al., 2003). For this reason, we assessed spongy moth population structure using two different sets of markers: (1) putatively divergent selected SNPs (high *F*_ST_), the “divergent set”; and (2) putatively neutral SNPs, the “neutral set”. To identify loci under putative divergent selection and to reduce the incidence of SNPs being incorrectly defined as outliers (i.e. false positive; Narum & Hess, 2011), three different genome scan methods were used: (1) a Bayesian method implemented in BayeScan v.2.1 (Foll & Gaggiotti, 2008), (2) the software OutFLANK, using a likelihood approach that identifies *F*_ST_ outliers by inferring a distribution of neutral *F*_ST_'s using likelihood on a trimmed distribution of *F*_ST_ values (Whitlock & Lotterhos, 2015), and (3) a multivariate approach available in the R package *pcadapt* (Luu et al., 2017; see details in Data S1). SNPs identified as divergent by at least two methods were included in the divergent set.

We also tested whether any of the putatively divergent SNPs were in sequences matching known genes. For each SNP, we extracted from the reference genome a 200‐bp sequence containing the SNP at its center, which we then used as query in a blastx search conducted against lepidopteran sequences in GenBank's nr database. A blastx hit was considered significant if its *E*‐value was ≤10^−6^. Minor allele frequency per population was then plotted against sampling longitude for each divergent SNP in order to identify clinal patterns of allele frequency shifts.

### 2.6 Range‐wide population diversity and structure

To assess population genomic diversity, we computed the unbiased gene diversity (unbiased *D* or *uHe*) based on the neutral dataset using the function *Hs()* of the R package *adegenet* (Jombart & Ahmed, 2011). As sample size was low for some populations (<10), subsampling techniques were used to investigate the effect of the number of individuals on unbiased *D* estimates (see Data S2). Results revealed that 15 individuals are sufficient to recover accurate within‐population unbiased gene diversity estimates. Thus, unbiased *D* variation among subspecies and geographic regions is reported only for populations with a sample size ≥15 moths (44 locations).

The extent of pairwise population differentiation was quantified based on the neutral set through computation of the unbiased estimator θ (Weir & Cockerham, 1984), using the *fst_WC84* function of the *assigner* R package (Gosselin et al., 2020). *F*_ST_ confidence intervals were estimated by running 100 bootstraps of markers (resampling with replacement), and on populations featuring >15 moths, the sample size that yields accurate estimates (Willing et al., 2012).

Population structure was first assessed using principal component analysis (PCA), which allows visualization of the overall variability among individuals and populations. As PCA does not accept missing data, the latter were replaced with the mean allele frequencies calculated on the entire dataset using the R function *scaleGen* (R package *adegenet*; Jombart et al., 2010). Population structure was also inferred using a model‐based method employing a maximum‐likelihood approach implemented in ADMIXTURE v1.3.0 (Alexander et al., 2009) and a *k*‐means algorithm implemented in the *find.clusters* function of the *adegenet* R package (Jombart et al., 2010). We ran ADMIXTURE with a cross‐validation procedure for a number of groups *K* varying from 2 to 30. For each *K* value, calculations were repeated 10 times, using different random seeds to assess the stability of the estimate. The most likely numbers of *K* populations were identified as being the ones exhibiting the lowest cross‐validation error compared to other *K* values. The number of populations was also assessed using a *k*‐means method based on the Hartigan‐Wong algorithm implemented in the *kmeans* function (*stats* R package). The *find.clusters* function (*adegenet* R package) was used to run sequentially the *k*‐means method with an increasing number of *K* values (from 2 to 30), and the optimal number of groups was identified based on the Bayesian information criterion (BIC; lowest value). The *find.clusters* function was also run 10 times with different random seeds. The missing values present in the data set were replaced with the mean allele frequency calculated on the entire dataset. For each *K* groups identified by the maximum‐likelihood approach and the *k*‐means method, the existence of lower‐level structures was explored using Discriminant Analysis of Principal Components (DAPC; Jombart et al., 2010), which has the property of effectively highlighting genetic differentiation among groups while overlooking within‐group variation (Jombart et al., 2010). The pertinent number of principal components retained for DAPC analysis was submitted to a cross‐validation test using the R function *xvalDapc* (R package *adegenet*; Jombart et al., 2010). Analyses of molecular variance (AMOVA, Excoffier et al., 1992) were performed in Arlequin v3.5.2.2 (Excoffier & Lischer, 2010) to assess the significance of the sub‐groups identified by DAPC analyses. Finally, the assignment of individuals to a given population was computed using the R function *predict.dapc* (R package *adegenet*; Jombart et al., 2010), which is based on the outcome of the DAPC analysis (see Data S5 for additional details). The PCA, ADMIXTURE and *k*‐means analyses were conducted on both the neutral and divergent datasets.

To infer relationships among populations and identify potential “migration events” (i.e., occurences of gene flow) among them, a population graph tree was built using the software TreeMix (Pickrell & Pritchard, 2012). TreeMix uses large numbers of SNPs to estimate the historical relationships among populations, using a graph representation that allows both population splits and migration events. This method uses the covariance of allele frequencies between populations to build a maximum likelihood tree relating populations to their common ancestor, and allows adding migration events between populations to improve the model fit to the data. Each analysis was run with the sequential addition of 1 to 10 *m* (migration events). For each value of *m*, 10 independent runs were performed with random orders of input populations (different starting point for the calculations; option—seed) to evaluate the convergence of the tree topology and migration events (Pickrell & Pritchard, 2012). In addition, the migration weights (i.e. *w*, genetic contribution from population X to population Z provided in %) and their standard error were estimated by jackknife resampling (option—se; block of 1 as SNPs are assumed to be unlinked) and a *p*‐value for each migration event was calculated. The optimal number of migration events was estimated using an ad hoc statistic (Δ*m*) based on the second‐order rate of change in the log likelihood (method Evanno) and implemented in the OptM R package (Fitak, 2021). Here, for the optimal number migration events, we considered the population tree displaying the highest maximum likelihood over 10 independent runs.

### 2.7 Subspecies tree estimation

To assess the relationship between the Caucasian/Middle‐Eastern population and the three recognized subspecies, we built a subspecies tree based on four or five individuals from each of the three subspecies plus the Caucasus/Middle East group (see Section 3) for a total of 18 moths (selected individuals are identified in Table S3). The choice of the individuals was based on their low level of missing data and their being geographically located outside the hybrid zones identified in the population structure analyses; North American specimens were not included in this analysis. The multi‐species coalescent method implemented in the BEAST2 add‐on package SNAPP v1.5.1 (Bryant et al., 2012) was used to infer subspecies tree topologies, along with posterior probabilities for each node of the tree. For this analysis, we used the 1528 polymorphic SNPs remaining after considering only the 18 moths we selected. The forward (*u*) and backward (*υ*) mutation rates, that is, the instantaneous rate of mutation from allele 0 to allele 1 and conversely, were fixed at 1. As initial state of the “coalescent rate” parameter, we used the default value of 10, but we subsequently estimated it (i.e., through sampling) in the course of running the MCMC chain. The priors for the speciation rate (λ) and the ancestral population's sizes (theta) were chosen to follow broad gamma distributions, as we do not assume strong a priori knowledge about the parameters (speciation rate: *α* = 2 and *β* = 200; the ancestral population's sizes: *α* = 1 and *β* = 250). We ran two independent MCMC chains for 3 × 10^6^ generations, with a pre‐burning of 40,000 generation length and sampling for parameters and trees every 100 generations. Using Tracer v1.7.1 (Rambaut et al., 2018), convergence and mixing of the MCMC chains were confirmed with an absence of trend in parameter traces, with high effective sample size (ESS) values for the different parameters (>200) and with similar parameter estimates for the two chains. We visualized the tree distribution using Densitree v2.2.6., which displays uncertainty in topology (Bouckaert, 2010).

## 3 RESULTS

### 3.1 SNP genotyping and filtering

We obtained an average of 83 million reads per library, and the Tassel 3.0 GBS pipeline identified an average of 6.83 million tags per plate, computed from 75 million reads. Of the 46,492 SNPs identified by Tassel 3.0 GBS, 2335 SNPs remained after applying the filtering procedure (Table S4). As expected for GBS outputs, the removal of missing data is the step that discarded the most SNPs (83% of initial SNP data set). The four SNPs excluded at the inter‐loci linkage disequilibrium filtering step were all in loci physically close to one another in the reference genome (from 76 to 3912 bp apart).

### 3.2 Outlier detection

**FIGURE 2:**
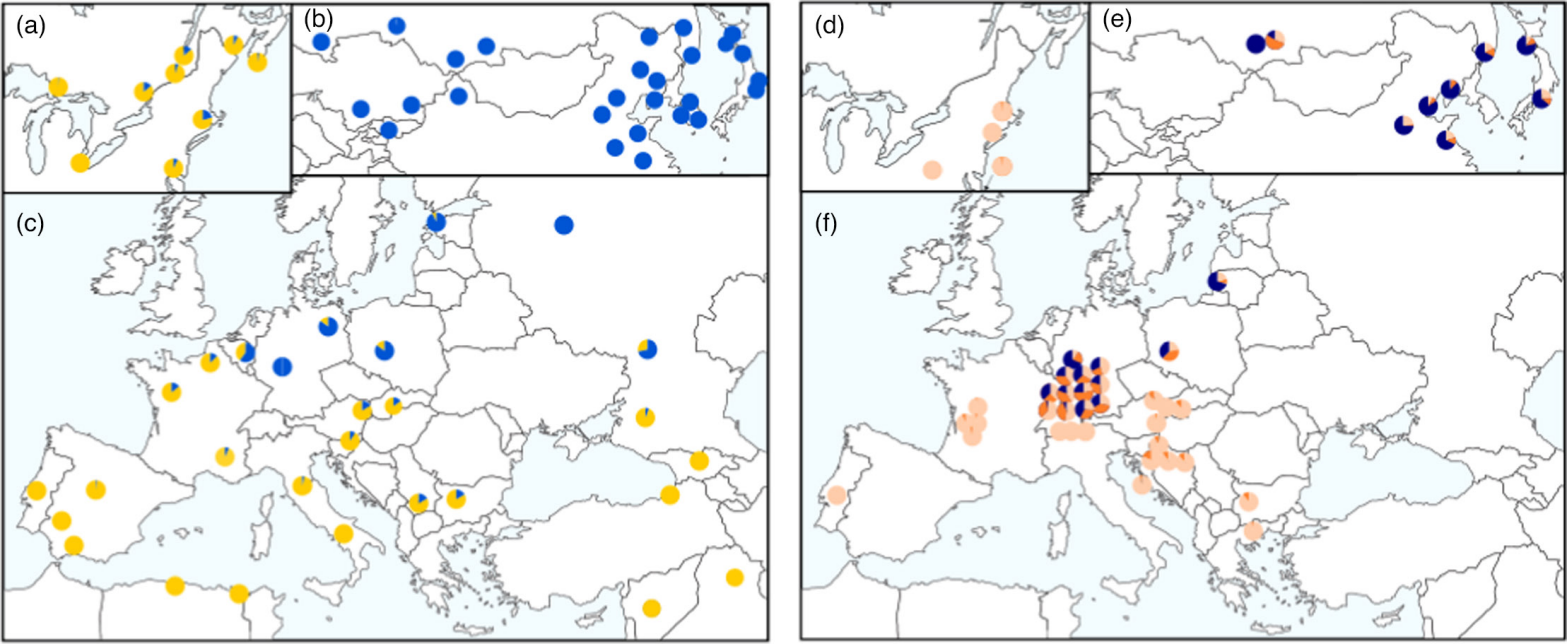
Geographic distribution of *Lymantria dispar* female flight capability mirrors the allelic frequency distribution of a highly divergent SNP. Left panel: Allelic frequency of SNP “Lda_tig00005865_39025”, located in the *ionotropic glutamate receptor, kainate 2‐like* gene, in North America (a), Asia (b) and Europe (c). Right panel: Proportion of flight‐capable, gliding‐capable (flight lacking upward displacement; gentle fall with wing flapping) and flightless spongy moth females, shown respectively in dark purple, dark orange and light orange, among populations in North America (d), Asia (e) and Europe (f). The data used to generate the right panel are from Keena et al. (2008).

Using three different genome scan methods, 77 SNPs (3.3%) were deemed to be putatively under divergent selection, while 2125 SNPs were considered neutral (see Data S1). Blastx analysis of the 200 bp sequences containing putatively divergent SNPs at their center provided a total of 20 hits with an *E*‐value ≤10^−6^ (Table S5). Among these different hits, a SNP (Lda_tig00005865_39025) located in the *ionotropic glutamate receptor of kainate 2 subtype* (Grik2 or Glur6) gene revealed a clinal pattern of allele frequency mirroring variation in female flight capacity observed across the geographic range of the spongy moth (Figure 2; Keena et al., 2008). Tblastx analyses against the reference genome of the silkworm, *Bombyx mori* (KAIKOBase v.4.0.0, https://kaikobase.dna.affrc.go.jp/index.html), indicated that the SNP and its associated scaffold (Lda_tig00005865) map to an autosome (chromosome 23), an observation that is consistent with conclusions drawn from the results of crossing experiments (Keena et al., 2007) that found no evidence of sex‐linkage of the female flight capability trait in the spongy moth.

### 3.3 Population genetic diversity and distances

**FIGURE 3:**
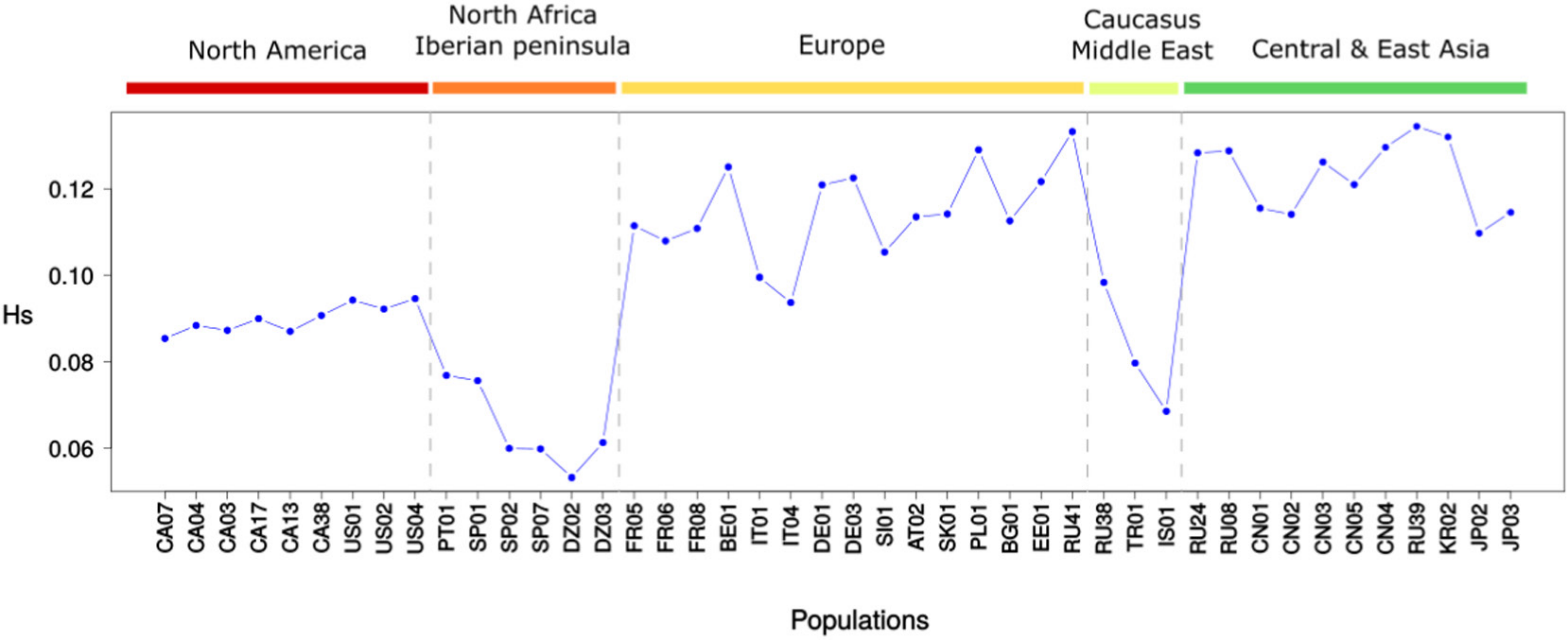
Gene diversity (*unbiased D*) computed for *L. dispar* populations featuring a sample size of at least 15 individuals (44 populations).

Gene diversity indices (unbiased *D*) varied notably across the spongy moth's range (Figure 3). The lowest values were observed in two population groupings, namely Iberian Peninsula/North Africa (0.065) and Caucasus/Middle East (0.082). In comparison, those from North America displayed somewhat higher values (0.090), but significantly lower than those of European (0.115) and Asian (0.123) populations (ANOVA, *F*_2,32_ = 32.72, *p* < 0.001), which were not significantly different from one another (Tukey's test, *p* = 0.056). However, an overall decline in gene diversity was observed from the Russian Far East (e.g., RU39) to Western Europe, with values lower for Japan than for continental East Asia (Figure 3). With the exception of the Balkan Peninsula, populations around the Mediterranean (Iberian, North African, Middle East/Caucasus and Italian), displayed lower genetic diversity than other Eurasian populations.

Average population differentiation was high, with a mean pairwise *F*_ST_ of 0.1917, while individual pairwise *F*_ST_ values (Figure S2) ranged from 0.0002 (CN01 vs. CN02, both in China) to 0.4847 (DZ02, Algeria vs. JP03, Japan). Bootstrapping‐based confidence intervals indicated that only three pairwise *F*_ST_ values were not significantly different from 0 (i.e., representing statistically indistinct populations). Two of these pairwise comparisons involved populations from central Europe (AT02 vs. SK01 and BG01 vs. SK01), while the third one involved populations from China (CN01 vs. CN02). Relative to the three established subspecies in Eurasia (Figure 1; Pogue & Schaefer, 2007), North American and Caucasus/Middle Eastern populations displayed genetic distances varying from 0.21 to 0.37 and from 0.17 and 0.39, respectively; these *F*_ST_ ranges are similar to those observed among the recognized *L. dispar* subspecies (0.20–0.34; Figure S2B).

### 3.4 Population structure

**FIGURE 4:**
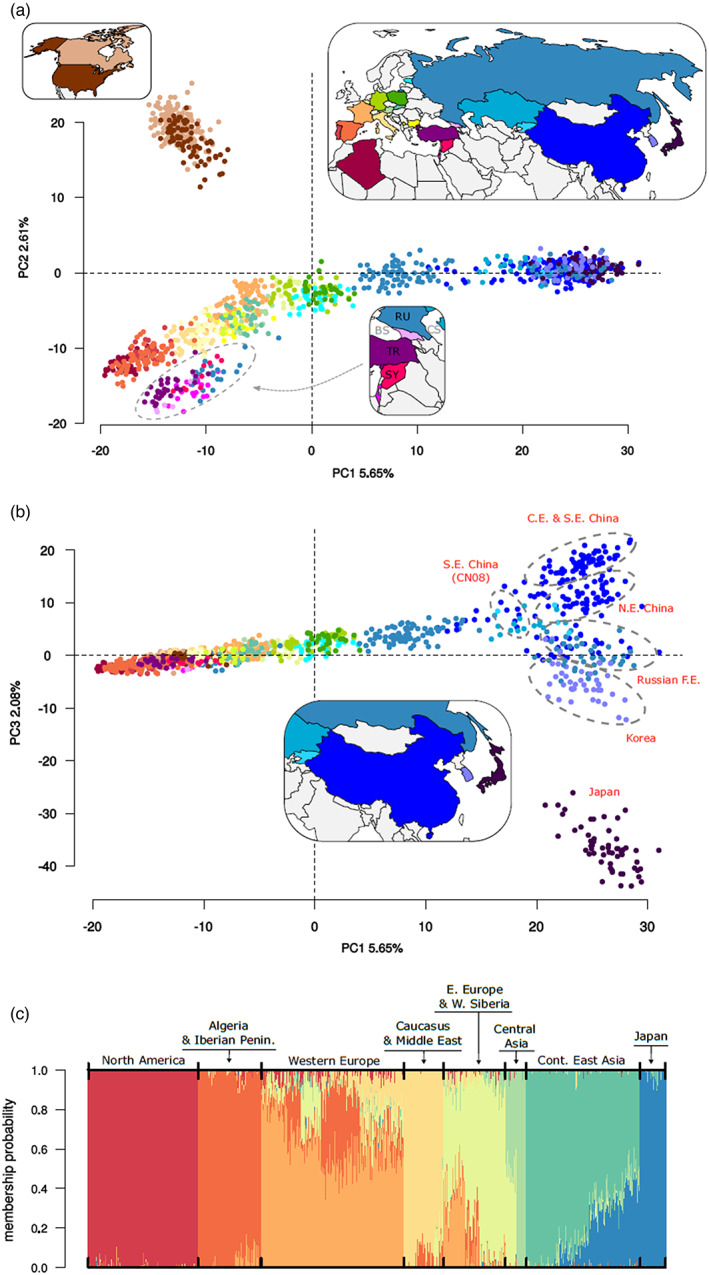
Population structure analysis. Principal component analysis (PCA) based on 2125 neutral SNPs derived from 1288 *L. dispar* individuals. (a) PC1 versus PC2 plot. Individuals (dots) are colored according to their country of origin (25 different countries). (b) PC1 versus PC3 plot. The dotted ellipses indicate populations from (i) Korea (ii) the Russian Far East and extreme North‐Eastern China (CN04), (iii) North‐Eastern China and (iv) central‐eastern and South‐Eastern China, and (v) the southernmost Chinese population (CN08). (c) Barplot showing probability of membership of each sample to each of eight geographic clusters, as computed by ADMIXTURE. Samples along the x axis are ordered from west to east according to their longitudinal coordinates, with possible exceptions for some of the more southerly samples from the Caucasus/middle‐east, which were placed between those of Western and Eastern Europe.

A principal component analysis (PCA) performed on the 2125 neutral SNP set revealed a west‐to‐east gradient (PC1 5.65%) in Eurasia and northern Africa, with populations from the Iberian Peninsula and Algeria seen at one end of the gradient and populations from far east Asia observed at the opposite end (Figure 4A). The second major feature was a clear separation of North American populations from the other populations along the PC2 axis (2.61%). The second axis also revealed a cluster of populations from the Caucasus (Georgia and south‐western Russia) and the Middle East (Turkey, Israel and Syria) as distinct from those in Europe. Plotting of PC1 against PC3 (2.08%) further revealed how Japanese populations form a distinct cluster relative to other Asian populations (Figure 4B) and showed geographic cline differentiation among other far‐east Asian populations, namely (i) Korea, (ii) the Russian Far East and northernmost eastern China (CN04), (iii) north‐eastern China and (iv) central‐eastern and south‐eastern China. Surprisingly, the southernmost population in China (CN08) revealed a close genetic proximity with populations from Central Asia and Siberia (Figure 4B). The PCA computed on the divergent SNP set revealed a similar pattern of population structure, except that variances explained by PC2 and by PC3 in the neutral dataset were inversed in the divergent data set (Figure S3).

**FIGURE 5:**
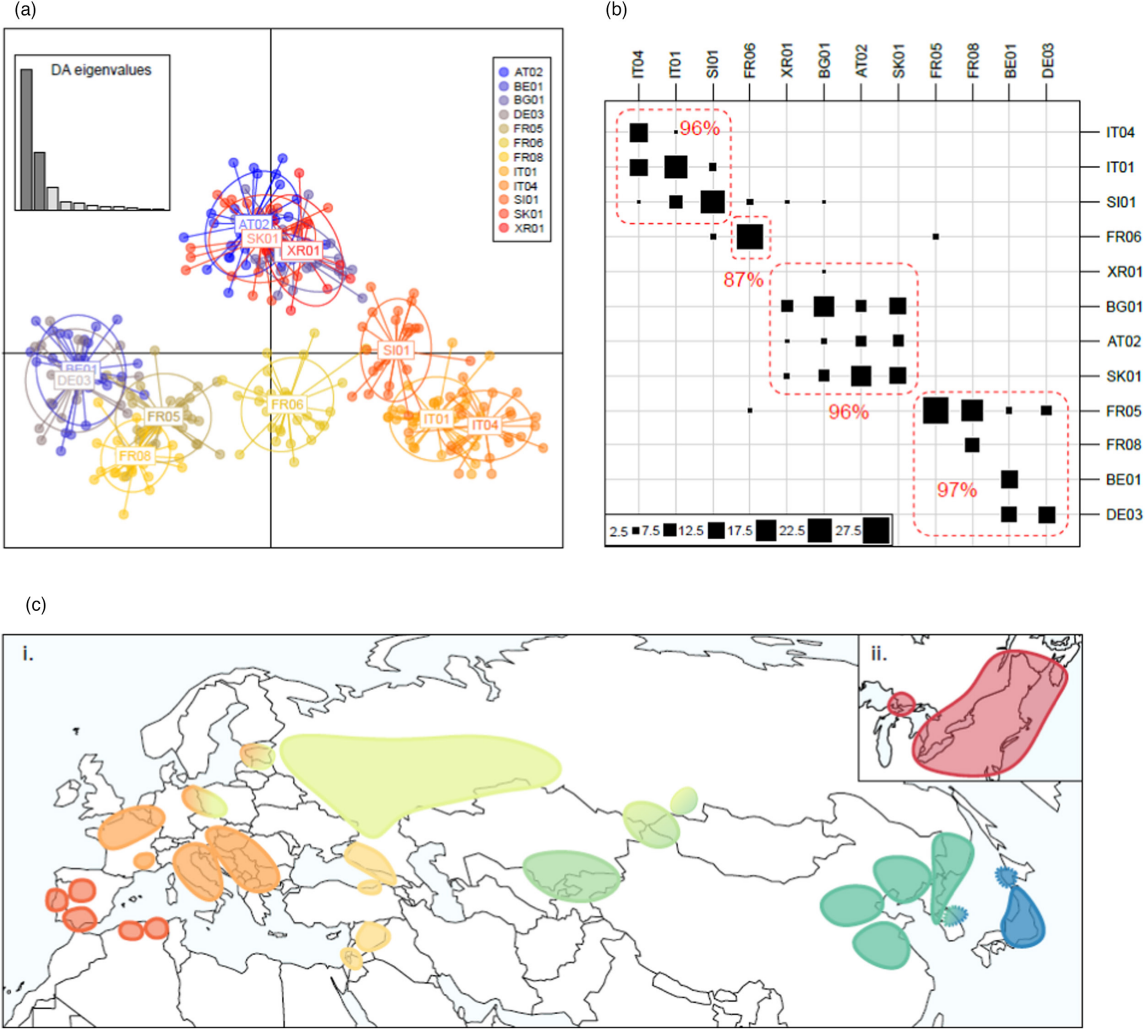
Fine population structure explored with (a) DAPC and (b) assignment analysis in Western and Central Europe as an example (analyses for other regions may be found in Data S5). The DAPC scatter plot (a) presents the individuals as dots, the populations as inertia ellipses and the eigenvalues in an inset. The assignment results (b) are presented in the form of a contingency table where the columns correspond to original populations of the individuals, while rows correspond to inferred populations. The position and size of the black squares indicate which populations the individuals were assigned to (according to maximum posterior probability of membership) and their number; the red dotted lines delimit the subgroups, and the figures in red show the percentage of correct assignment. We considered that a subgroup (one or several populations) was well supported when 80% of its individuals attained a posterior probability of membership ≥0.8. In (c), the map shows the 28 well supported subgroups identified in (i) Eurasia and (ii) North America. The two Asian groups identified with a dotted line in (i) reached nearly 80% of correct assignment, when the highest posterior probability of membership was used as criteria of correct assignment instead of the 0.8 threshold.

Based on neutral SNPs, the *k‐mean* methods implemented in the *find.clusters* function indicated that the minimum likely number of genetically distinct clusters was *K* = 8 or 9 (lowest BIC median and lowest BIC values, respectively) (Figure S4). With the maximum‐likelihood approach implemented in ADMIXTURE v1.3.0, the minimum number of groups varied between *K* = 9–12, but analysis of the membership coefficient revealed the presence of ghost clusters, i.e., groups in which no individual achieved a membership coefficient *Q* > 0.5 (Guillot et al., 2005). As suggested by Puechmaille (2016), we corrected the number *K* of clusters by removing those considered spurious, which resulted in a *K*_corrected_ = 8–10. Thus, both analyses identified a minimum of eight groups: (1) North‐America, (2) Iberian peninsula and Algeria, (3) western and central Europe (France, Belgium, western Germany, Italy, Slovenia, Austria, Slovakia, Kosovo, Bulgaria), (4) Caucasus and Middle East, (5) northern Europe (north‐east Germany, Poland, Estonia) and western Russia up to the Novosibirsk region, (6) eastern Central Asia (Kazakhstan, Kyrgyzstan, Altay region in Russia and China), (7) east continental Asia and (8) Japan (Figure 4C). The ADMIXTURE plot highlighted the presence of blurred edges of some adjacent geographic groups (from Western Europe to continental East Asia), confirming PCA results of a gradual genetic cline through much of the spongy moth range. Regarding the outlier SNP set, the groups identified by both methods agreed with those found with the neutral dataset (see Data S3). Not surprisingly, the heatmap and dendogram based on pairwise *F*_ST_ values (Figure S2) separated samples according to the same geographic groups identified by the *k*‐mean methods and ADMIXTURE, using the neutral SNP dataset. For each of the 8 groups identified, AMOVA analysis indicated that at least one population or group of populations was significantly different from the others (*p*‐values ≤0.01). The DAPC and assignment analyses carried out in each of the *K* = 8 groups yielded 28 subgroups across the species' range (Figure 5; Data S5), in which the percentage of correct assignment of an individual to its population of origin averaged 95%.

**FIGURE 6:**
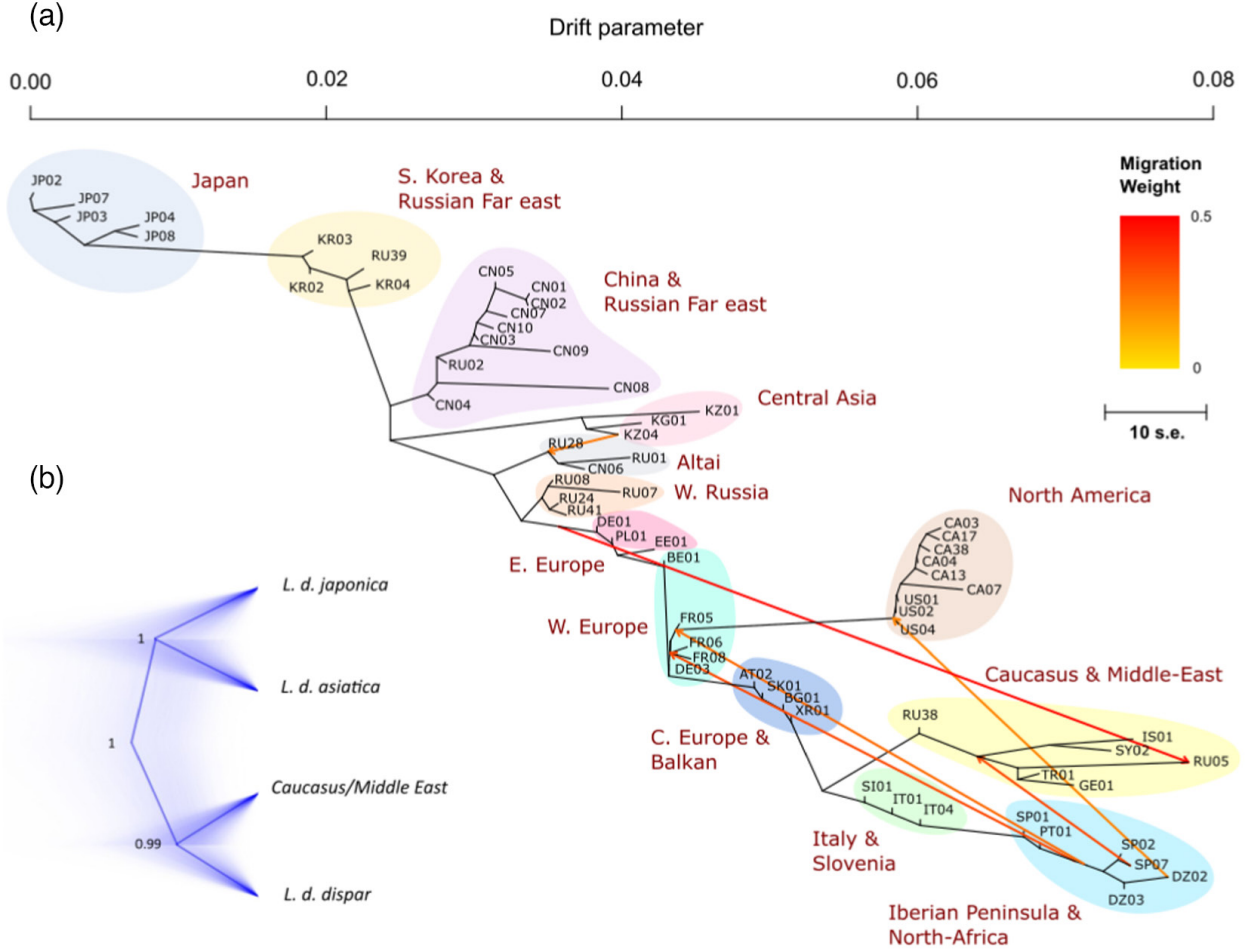
Population and subspecies trees. In (a), maximum likelihood population graph tree generated by TreeMix, with samples from a Japanese population (JP02) used to root the tree (see materials and methods for details). Arrows denote ‘migration events’ (likely occurrences of gene flow) between populations during their evolutionary history. Inclusion of these migration events improved model fit to the data. Only the horizontal length of branches has interpretive value in this graph and are proportional to the evolutionary change (i.e., the drift parameter); vertical lengths of branches are adjusted to optimize the graphical representation of the tree. In (b), genome‐wide‐SNP‐based spongy moth subspecies tree generated using a coalescent method implemented in the BEAST2 add‐on package SNAPP v1.5.1. Four or five specimens of each recognized subspecies (total of 18) were selected from regions where subspecies designation is considered certain (*L. dispar dispar*: Western Europe; *L. dispar asiatica*: Continental East Asia; *L. dispar japonica*: Main islands of Japan). Samples from the Caucasus/middle‐east were included in this analysis to assess their placement relative to the three recognized subspecies, and to compare the SNAPP tree topology with that obtained for mtDNA‐based trees (e.g., Djoumad et al., 2017). The choice of the individuals was based on their low level of missing data.

We conducted several iterations of the TreeMix‐based computation aimed at generating a population tree, using different East Asian spongy moth populations to root the tree, with no impact of the latter parameter on the overall inter‐population relationships depicted by the tree. The optimal population tree shown here, rooted with a population from Japan (JP02), explained 97.4% of the variance in relatedness among populations and inferred six ‘migration events’, all with *p*‐values ≤10^−9^ (Figure 6A; see Data S4 for an example of a tree with an alternative root). The high level of explained variance indicates that the tree adequately models relatedness among populations, with some possible exceptions (e.g., Algerian population), as suggested by the model's residual plot (Figure S5). The tree largely recapitulated the relationships already found among populations in the above analyses (Figure 4), that is, a population genomic structure that follows an east–west geographic gradient, with distinct clusters formed by the North American and Caucasian/Middle‐Eastern populations. This analysis also indicated that North‐American populations originated from a location on the west coast of France, in the Vendée region (FR05). Regarding migration events, the analysis seems to confirm cases of admixture also identified by ADMIXTURE analysis in populations at the edges of the different population clusters (see above and Data S5). However, a migration event was unexpected: the North‐American populations would trace about 21% of their ancestry to an Algerian population (DZ02, *p*‐value = 6.92 × 10^−9^). In view of the weaker modelling success of the Algerian population and the inferred French origin of the North American population, we interpret this apparent gene flow as an artefact associated with the genetic proximity of French and Iberian/Algerian populations, instead of a direct contribution of Algerian genes to the North American population.

### 3.5 Subspecies tree estimation

The multi‐species coalescent method implemented in SNAPP v1.5.1 yielded a tree supporting a first dichotomy between European and Asian lineages. Then, the Asian lineage split into the two recognized Asian subspecies, *L. d. asiatica* and *L. d. japonica*, whereas the European lineage separated into *L. d. dispar* and the Caucasus/Middle East group. All nodes were well supported with posterior probability values ≥0.99 (Figure 6B).

## 4 DISCUSSION

Based on a large collection of genome‐wide SNPs obtained from contemporary specimens, the present study provides a thorough characterization of spongy moth population structure across its entire geographic range, achieving an unprecedented degree of resolution for this species. The analyses we conducted raise new questions about the spongy moth's evolutionary history and about the current three‐taxa subspecies classification system. In addition, our study lays the foundation for the development of a reliable, genomics‐based geographic source identification assay for this destructive pest (e.g., AmpliSeq panels).

### 4.1 Population structure of *L. dispar*

The native range of the spongy moth stretches from the Japanese archipelago all the way to western Europe and parts of northern Africa, thereby covering most of Eurasia (Pogue & Schaefer, 2007). This Palearctic distribution was extended to northeastern North America when individuals were accidentally introduced in Massachusetts in 1868–1869, from where they successfully spread to neighboring US states and Canadian provinces (CFIA, 2022; Forbush & Fernald, 1896; USDA‐APHIS, 2022). Based on 2125 neutral SNPs genotyped for 1256 moths, we were here able to distinguish, within this vast range, eight major geographic groups that could be further divided into 28 subgroups (see Section 3.4 for details). As a result, genetic differences were identified at a population or regional (~500 km) scale in the species' native range, thus providing the most detailed and complete characterization of spongy moth population genomic structure to date. Such a fine population structure in the native range is likely explained by this species' limited dispersal capacity, which slows genetic homogenisation caused by gene flow (Bohonak, 1999). Indeed, whether it be at the larval stage through ballooning or at the adult stage by ascending flight, mean dispersal distances do not seem to exceed 100 m (Cameron et al., 1979; Mason & McManus, 1981; Minott, 1922) and 10 km (Baranchikov, 1988; Liebhold et al., 2008; Srivastava et al., 2021), respectively. In comparison, the spruce budworm, *Choristoneura fumiferana*, displays weak spatial genetic structure across its North American continental range, likely as a consequence of the long‐distance migrations its adults undertake (Lumley et al., 2020).

In continental East Asia, our analyses recovered five genetically different groups whereas earlier work led to the identification of only two to four such clusters (Chen et al., 2013, 2016; Kang et al., 2017; Keena et al., 2008; Wu et al., 2015; Zhao et al., 2019; Zuo et al., 2019). These five groups displayed genetic distances that mirrored their geographic locations in continental east Asia, but, interestingly, the southernmost Chinese population (CN08 in the Anhui province) revealed genetic proximity with moths from central Asia and Siberia located more than 3000 km away. Chen et al. (2016) observed a similar pattern with COI barcode sequences from a portion of moths sampled within ~100 km of our CN08 population, while other specimens from the same location had typical *L. d. asiatica* COI barcode signatures. Such genetic pattern could be a legacy of gene flow between Chinese and central Asian populations, through either natural or human‐aided movement. Our analyses also provided finer resolution among geographic populations in Europe than previously achieved (Lacković et al., 2018; Wu et al., 2015, 2020). It should be noted that boundaries between some of these groups correspond to topographic barriers known to limit gene flow between animal populations, for example, the Pyrenees and Dinaric and Carpathian Alps (Hewitt, 2000; Lacković et al., 2015; Schmitt & Varga, 2012). Based on morphological, mitochondrial and nuclear data, previous investigations have led to the suggestion that spongy moths in the Mediterranean region (Italy, Spain and North Africa) stand out from other European populations (Goldschmidt, 1940; Wu et al., 2015, 2020). The genetic diversity and distance indices (*unbiased D*, *F*_ST_) and population structure analyses (PCA, ADMIXTURE, TreeMix) presented here indeed confirmed the distinct status of spongy moths from the Iberian Peninsula and North Africa, while the Italian/Slovenian populations displayed less pronounced distinctiveness. Based on these results, the Pyrenees seem to be a more important barrier than the Alps to spongy moth gene flow. In contrast with East Asia and Europe, western and central Russia (here, the region extending from Moscow to Novosibirsk), which covers >3000 km longitudinally, revealed limited spatial genetic structure. An earlier study (Martemyanov et al., 2019) based on COI sequences also pointed to a low level of genetic structure in an area extending over 1500 km east of the Urals. This genetic homogeneity suggests the existence of gene flow on a broad scale in this region. Based on the observation of larvae far from forest edges and the spread of a recent outbreak in the direction of dominant winds during larval stages in the spring, Martemyanov et al. (2019) suggested that larval ballooning could play a significant role in this dispersal.

Regarding the Caucasian/Middle‐Eastern populations, both genetic distance estimates (*F*_ST_) and population structure analyses (PCA, ADMIXTURE, TreeMix) clearly highlighted the genetic singularity of spongy moths in this region. Previous studies based either on mitochondrial sequences (Djoumad et al., 2017; Zahiri et al., 2019) or on a combination of mitochondrial and nuclear loci (Wu et al., 2020) led to similar conclusions. The work of Zhang et al. (2019), however, challenged these conclusions, with a PCA analysis based on nuclear genome‐wide sequences that could not distinguish two Iranian specimens from European spongy moths. However, the same individuals were clearly distinct from other spongy moth lineages, based on their mitochondrial genomes. Such mito‐nuclear discordance suggests that the Iranian moths analysed by Zhang et al. (2019) possessed a European nuclear genome, while displaying Caucasian/Middle‐Eastern mitogenome introgression. The genetic distinctiveness of the Caucasian/Middle‐Eastern spongy moth population reported here is likely the outcome of geographic isolation caused, in part, by physical barriers such as the Caucasus Mountains. The same type of singularity has been observed in other Holarctic Lepidopteran species (Hegna et al., 2015; Maresova et al., 2019).

### 4.2 Population structure and origin of the moths introduced in North America

The North American spongy moth population showed singular allelic frequencies and low genetic structure and diversity, as expected after a strong founder effect followed by rapid population expansion (Dlugosch & Parker, 2008). Interestingly, our westernmost population (CA07), close to the expansion front in the province of Ontario (Canada), stood out from the remaining, genetically homogeneous North American populations (Data S5). In previous work, spongy moth populations at range edge also exhibited unique genetic features such as elevated frequencies of alleles otherwise rare in North‐America or alleles not detected in other part of the species' distribution (Friedline et al., 2019; Wu et al., 2015). The distinctiveness of populations near the range edge may be the outcome of allele surfing, where a rare or common allele surfs the wave of an expanding population front and reaches high frequency in newly colonized areas (Excoffier et al., 2009).

The question of the exact origin of the spongy moths accidentally introduced by French entomologist Leopold Trouvelot in Massachusetts 150 years ago has often resurfaced in the literature. It has typically been assumed that the intruders, like Trouvelot, were from France, a claim that found some genetic support in mitochondrial and microsatellite data (Harrison et al., 1983; Harrison & ODell, 1989; Wu et al., 2015). Other analyses reported that some North American moths had mtDNA haplotypes closer to those of specimens from Germany and Sardinia (Bogdanowicz et al., 2000; Wu et al., 2015). However, the present TreeMix and PCA analyses, based on our panel of genome‐wide SNPs, confirmed a French origin of the introduced spongy moths, pointing more precisely to the northwestern region of France (FR05—Vendée).

### 4.3 Geographical limits of the three *L. dispar* subspecies

As the present work strongly suggests that spongy moth populations form eight main genetically distinct groups across the species' range, the question arises as to whether such spatial genetic structure is compatible with the current three‐subspecies classification system put forward by Pogue and Schaefer (2007) and widely adopted since then. In fact, our data seem more in line with the early classification of Goldschmidt (1934, 1940), who recognized seven subspecies or “geographic races” across Eurasia. This being said, Goldschmidt (1940) did not consider all of his seven subspecies “of equal value”, suggesting that the Pogue and Schaefer's subspecies classification may still prove useful from a practical standpoint. This classification is also, in large part, supported by mtDNA‐based phylogenies (e.g., Djoumad et al., 2017; Wu et al., 2015). As a consequence, we here take a close look at this system in the light of our data.

The delimitation of the three *L. dispar* subspecies was based on differences in morphology, dimorphism in female flight capability and geographic range. Thus, according to Pogue and Schaefer's revision of the genus *Lymantria*, *L. dispar japonica* is found in the main islands of the Japanese archipelago, whereas *L. dispar dispar* and *L. dispar asiatica* are found in Europe (plus North America) and continental Asia, respectively. The geographical limit between the latter two subspecies was considered to be the Ural Mountains in western Russia. In the present study, the spongy moths sampled in Japan (Honshu and western Hokkaido) were found to be genetically distinct from other populations (Figures 4B,C and 6A). A strong differentiation of Japanese populations was also reported by earlier studies based on analyses of microsatellite markers or 60 mitochondrial and nuclear loci (Wu et al., 2015, 2020). Thus, genetic data seem to confirm the geographic circumscription of the *japonica* subspecies to the main islands of the Japanese archipelago, as proposed by Pogue and Schaefer (2007), thus providing genetic validation of the geographical definition of this subspecies.

The situation seems far more blurred with respect to the *L. d. dispar*/*L. d. asiatica* geographic boundary in Eurasia, particularly in view of the fact that the type specimen for the *asiatica* subspecies comes from Kazakhstan, as opposed to east Asia (Korb & Pozhogin, 2012; Zahiri et al., 2019). For example, recent studies indicated that central Russian populations as far east as Novosibirsk display COI barcode and microsatellite genotypes of *L. d. dispar*, apparently shifting the subspecies boundary much to the east of the Ural Mountains (Martemyanov et al., 2019; Wu et al., 2015; Zahiri et al., 2019). From our nuclear genome‐wide SNPs, we could not identify a sharp boundary between the two subspecies; rather, we observed a cline from what is referred to as *L. dispar asiatica* in East Asia to *L. d. dispar* in Western Europe (excluding, here, the more southerly located Caucasus/Middle‐Eastern population). The existence of such a genetic cline was also suggested by the work of Wu et al. (2020), using a combination of mitochondrial and nuclear loci. Interestingly, female flight capability, the main biological trait used by Pogue and Schaefer (2007) to distinguish *L. d. dispar* from *L. d. asiatica*, also displays an east–west cline. Indeed, spongy moth females from East Asia are capable of strong directed flight, while those from southern and Western Europe are flightless and those from the hybrid zone show variable degrees of flight capability (Keena et al., 2008; see Figure 2D–F). The existence of such a cline makes the direct use of our data impractical to distinguish them in the hybrid zone. However, as the assignment analyses presented here show, our SNP dataset can be used for a fine‐scale identification of the geographic origin of a moth, including moths from the *dispar/asiatica* hybrid zone. Thus, SNP‐based source identification, together with geo‐referenced female flight capability data (Keena et al., 2008), could be used to determine whether an intercepted specimen poses a risk with respect to introducing the female flight capability trait into North America. Ideally, future work should focus on identifying the genes responsible for female flight capability and on selecting diagnostic SNPs for this trait, which could then be added to a molecular tool developed for source identification. In this regard, the present study identified a highly divergent SNP located in the *ionotropic glutamate receptor*, *kainite 2‐like* gene, with an allelic frequency distribution that mirrors that of female flight capability (Figure 2). Zhang et al. (2019) also identified nucleotide substitutions in this gene when comparing European and Asian spongy moth genomes. We are currently taking steps to assess the functional relationship between this gene and female flight capability.

Beyond the above considerations, the clear genetic distinctiveness of the North‐American and Caucasian/Middle Eastern populations raises the question of whether they deserve subspecies status. Subspecies delineation is a complex issue given that criteria used by scientists for this type of classification tend to differ according to the taxon being considered and the type of data on which the decision is based (genetic, morphology, ecology). Recently, Galtier (2019) proposed to use species or subspecies for which boundaries are widely accepted to establish threshold criteria that can then be used to assess species or subspecies status in other taxonomic groups. If we apply this approach to the spongy moth, mean genetic distances (*F*_ST_) among populations of the three established *L. dispar* subspecies can be used as genetic threshold for attribution of subspecies status to other populations. In the present study, North American and Caucasian/Middle Eastern populations displayed genetic distances with other populations similar to or greater than those observed among the three recognized *L. dispar* subspecies in Eurasia (populations in *dispar*/*asiatica* hybrid zone were not considered; Figure S2). Similar results were reported by Wu et al. (2020) for the North American population. Thus, from a genetic point of view, North American and Caucasian/Middle Eastern populations meet Galtier's (2019) criteria for being considered subspecies of *L. dispar*. Ultimately, biological attributes, such as morphology, ecology and behavior, should be examined to assess whether these presumed subspecies display other distinctive traits or meet the definition of cryptic subspecies. Indeed, little is known about their biological singularities beyond anecdotal accounts pertaining to possible differences in host tree preferences for the Caucasian/Middle Eastern population relative to others.

### 4.4 Evolutionary history of *Lymantria dispar*

The subspecies tree presented here, which is based on genome‐wide SNPs from 18 specimens (Figure 6B), points to a first separation between European and Asian lineages from the common ancestor of *L. dispar*. Thereafter, the European lineage gave rise to the *L. d. dispar* subspecies and the Caucasian/Middle‐Eastern populations, whereas the Asian lineage split into the two sister subspecies, *L. dispar japonica* and *L. dispar asiatica*. The phylogenetic relationships obtained here for the three recognized subspecies (*japonica*, *asiatica*, *dispar*) are in agreement with previous phylogenies conducted using mitochondrial markers (Djoumad et al., 2017; Wu et al., 2015; Zhang et al., 2019; Zhao et al., 2019), with the exception that the Caucasus/Middle Eastern population formed a sister group to *L. dispar dispar* (this study) as opposed to being basal to all *L. dispar* subspecies (mtDNA‐based studies). Thus, neither mitochondrial nor nuclear DNA phylogenies support Goldschmidt's (1934) hypothesis of a Japanese origin for *L. dispar*, given that *L. d. japonica* is not the basal taxon in these trees. Regarding the Caucasus/Middle Eastern populations, their position in our tree points to a case of mito‐nuclear discordance and suggests that the Caucasus/Middle Eastern region is unlikely to represent the evolutionary origin of *L. dispar*, as proposed by Zahiri et al. (2019), based on our nuclear genome data.

On the basis of the subspecies tree presented here (Figure 6B) and the patterns of genetic diversity estimates shown in Figure 3, we can begin to sketch the likely evolutionary history of *L. dispar*. High genetic diversity estimates are expected in zones representing the geographic origin of a species, as range expansions come with the loss of alleles and a reduction of heterozygosity due to successive founding events along the colonization route (Chuang & Peterson, 2016; Hewitt, 2000; Schmitt, 2007). Thus, a first dichotomy between the European and Asian lineages in the subspecies tree, combined with a trend for gene diversity estimates to be highest in Korean, Russian Far East, Chinese and Siberian populations, points to continental East Asia as the likely cradle of *L. dispar*. Then, the lower genetic diversity estimates that we observed in Japan, here and in previous studies (Wu et al., 2015, 2020), and the position of *L. d. japonica* as a sister group to *L. d. asiatica* in the subspecies tree, suggest that the ancestors of *L. d. japonica* gained access to Japan from continental east Asia via the Korean peninsula. A hypothesis that is also supported by the TreeMix tree (Figure 6A) and the genetic proximity of Korean and Japanese populations in our PCA and population structure analyses (Figure 4). Wu et al. (2015) proposed a similar scenario based on mitochondrial and microsatellite data, but a spread via Sakhalin has also been suggested (Zahiri et al., 2019). However, our data do not support the latter scenario as we did not detect genetic proximity between Hokkaido and Russian Far East populations, which would have otherwise suggested colonization of Japan through Sakhalin. Eventually, ADMIXTURE analysis revealed *L. d. japonica* introgression into continental Asian populations in the vicinity of southern Japan, this phenomenon likely resulting from secondary contacts between these populations via the Korean Strait.

In Europe, the gradual decline in genetic diversity estimates from western Siberia to western Europe (this study; Keena et al., 2008; Wu et al., 2015; Wu et al., 2020; Zuo et al., 2019) suggests a progressive expansion from the Asian cradle. The divergence time estimated from mitochondrial data for the European‐Asian split suggests that *L. dispar* has been present in Europe for a long time, although these estimates vary considerably (0.3 Mya, Wu et al., 2015; 1.1 Mya, Zahiri et al., 2019). Therefore, spongy moth populations in Europe have faced the alternation of glacial and interglacial periods during the Pleistocene, which certainly affected their geographic distribution. The lower gene diversity indices observed in the Iberian Peninsula/Algeria, the Caucasus/Middle East and, to a lesser extent, Italy, is consistent with a scenario where these southern, warmer regions were colonized during glacial periods, with mountain ranges subsequently acting as barriers to gene flow during the retreat of ice, isolating these populations. In addition, current spongy moth habitats in these regions are expected to be sparse and fragmented, thus amplifying population isolation. This is certainly true of Algeria (https://www.bestcitymaps.com/digital‐maps/algeria‐vegetation‐map/) and Israel (https://mfa.gov.il/MFA/AboutIsrael/Maps/Pages/Israel‐Forests.aspx), where we observed some of the lowest gene diversity indices in the present study (Figure 3).

## 5 CONCLUSION

Spongy moth populations show a remarkable degree of spatial genetic structure across the native species' geographic range, a feature that is likely associated with the insect's limited mobility. The elevated degree of population structure reported here will certainly facilitate the development of molecular tools, in the context of genomic biosurveillance (Hamelin & Roe, 2020; Roe et al., 2019), aimed at identifying the geographic origins of moths intercepted during ship inspections in North American ports. In addition, while our analyses confirmed circumscription of the *japonica* subspecies to Japan, they also pointed to the absence of a sharp geographical boundary (e.g., the Ural Mountains) between the *dispar* and *asiatica* subspecies in continental Eurasia. Furthermore, based on genetic distances, we suggest that the North American and Caucasian/Middle Eastern *L. dispar* populations potentially deserve distinct subspecies status. Regarding the phylogeography of *L. dispar*, while the present work suggests a continental East Asian origin for the species, we are pursuing this line of enquiry through a more detailed assessment of its evolutionary history, taking into account the spongy moth's two sister species, *L. umbrosa* and *L. albescens*, and comparing the histories revealed by nuclear and mitochondrial genomes.

## CONFLICT OF INTEREST

The authors have no conflicts of interest to declare.

## Supporting information


Appendix S1

Appendix S2

## Data Availability

Raw sequences and GBS genotypes are available through Dryad under doi: https://doi.org/10.5061/dryad.wwpzgmsp5.
