# Supplementary material for: Range‐wide population genomics of the spongy moth, Lymantria dispar (Erebidae): Implications for biosurveillance, subspecies classification and phylogeography of a destructive moth
Source: Evol Appl. 2023 Jan 13;16(3):638–56. doi: 10.1111/eva.13522 (PMC10033852; doi:10.1111/eva.13522)

**Supplemental Information for:**

**Range-wide population genomics of the spongy moth, *Lymantria dispar* (Erebidae): implications for biosurveillance, subspecies classification and phylogeography of a destructive moth.**

**Table of Contents:**

| **Figure 1** | Page 2 |
| --- | --- |
| **Figure 2** | Page 3 |
| **Figure 3** | Page 4 |
| **Figure 4** | Page 5 |
| **Figure 5** | Page 6 |
| **Supplemental information 1** | Page 7 |
| **Supplemental information 2** | Page 12 |
| **Supplemental information 3** | Page 15 |
| **Supplemental information 4** | Page 17 |
| **Supplemental information 5** | Page 19 |


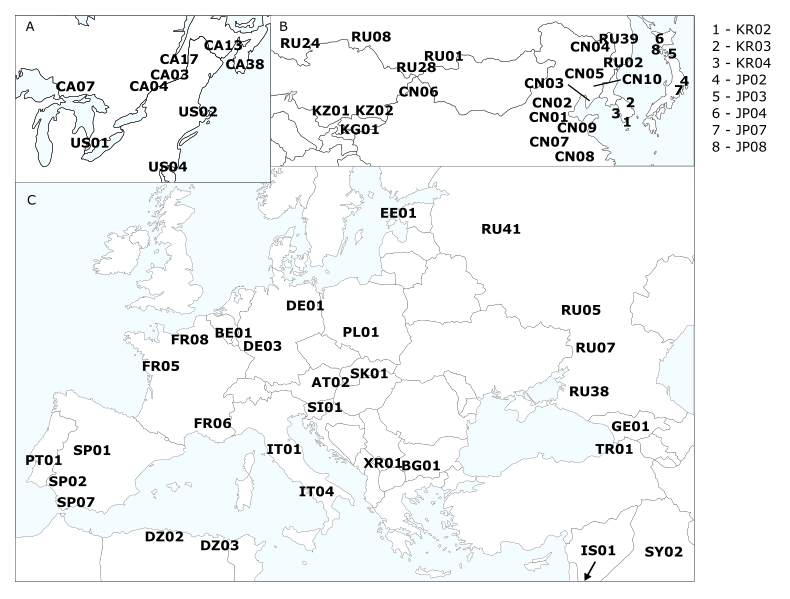


**Figure S1.** Sampling locations identified by their abbreviated names in (A) North America, (B) Asia and (C) Europe, North Africa and the Middle East.


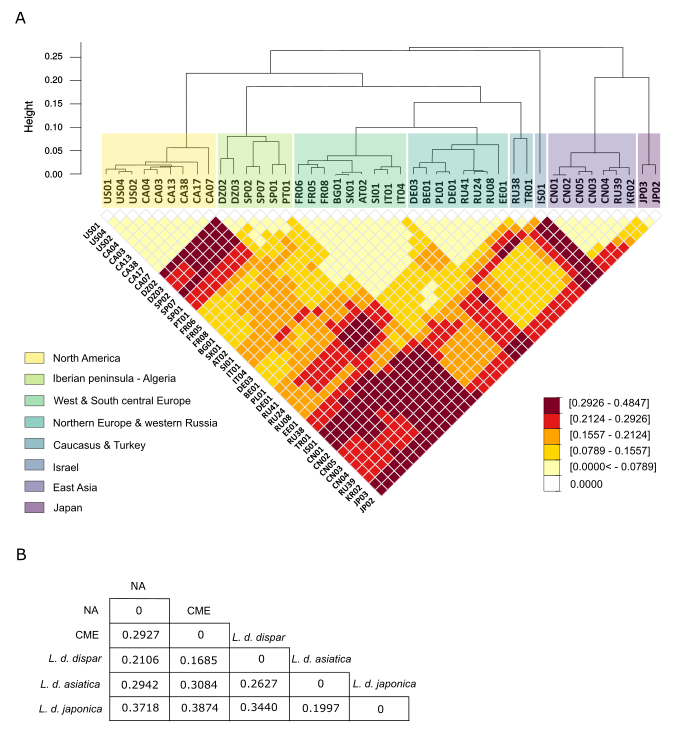


**Figure S2.** (A) Dendogram and heatmap based on pairwise *F_st_* values among 44 *L. dispar* sampling locations. (B) *F_st_* values among recognized subspecies, North-American (NA) and Caucasian/Middle Eastern (CME) populations. For *L. d. dispar* and *L. d. asiatica*, the populations considered for *F_st_* calculations are outside the hybrid/cline zone i.e. the calculations does not include the populations PL01, DE01, RU41, RU24, RU08 and EE01.


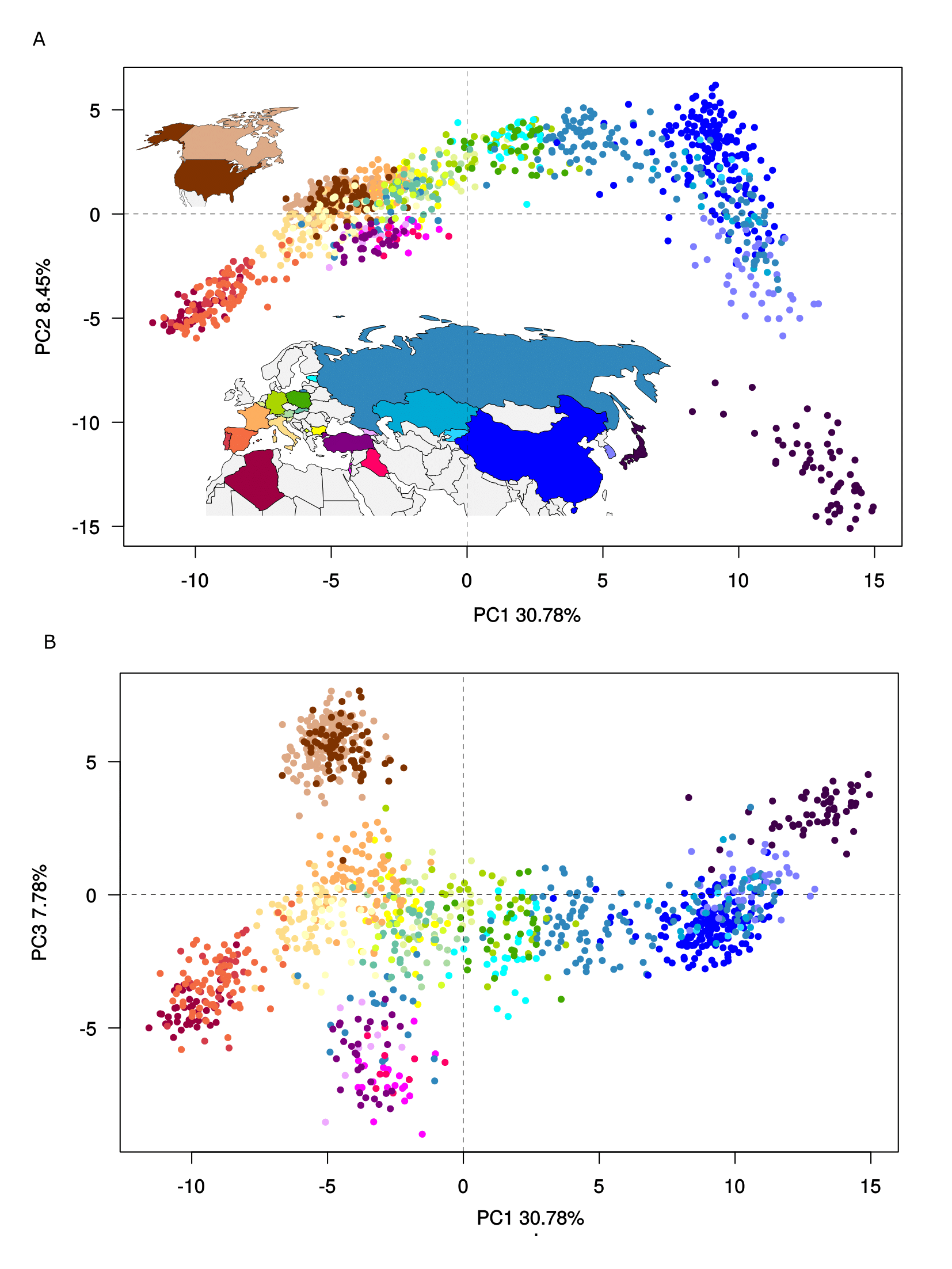


**Figure S3.** Principal component analysis (PCA) based on 77 divergent SNPs derived from 1288 *L. dispar* individuals. **(**A) PC1 *vs* PC2 plot and (B) PC1 *vs* PC3 plot. Individuals (dots) are colored according to their country of origin (25 different countries).


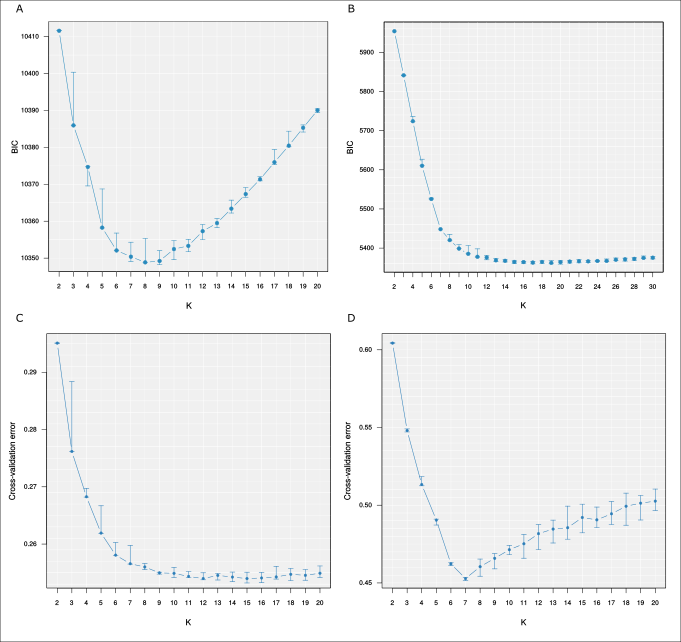


**Figure S4.** Bayesian information criterion (BIC; *k-mean* methods) and cross validation error scores (ADMIXTURE analysis) for various *K* numbers of groups for (A, C) the 2125 neutral SNP dataset and (B, D) the 77 divergent SNP dataset. For each *K* value, calculations were repeated 10 times, using different random seeds to assess the stability of the estimate. Each dot represents the median of the values obtained for the 10 replicates; each vertical bar shows the range of values. The most likely numbers of *K* populations were identified as being the ones exhibiting the lowest values compared to other *K* values.


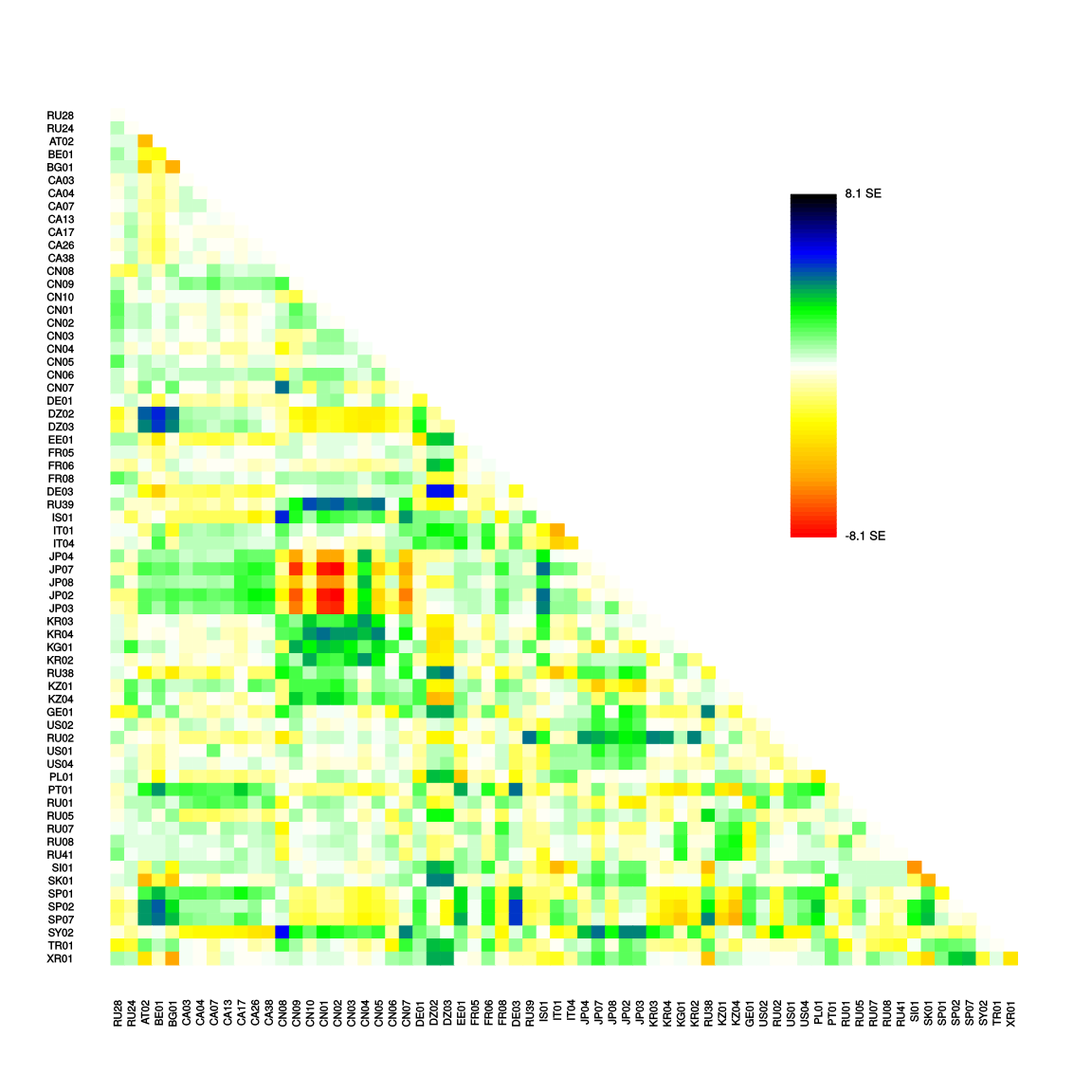


**Figure S5** Model residual plot of the maximum likelihood tree with six “migration events” inferred by Treemix. The plot presents the scaled residuals i.e the residual covariance between each pair of populations divided by the average standard error across all pairs. The highest positive residuals indicate pairs of populations not well modelled and for which the fit might be improved by adding a migration event between them.

**Supplementary information 1: Detection of loci under putative selection**

*Material and Methods*

Three different genome scan methods were employed to identify SNPs putatively under divergent selection (high *F*_st_). First, we used a Bayesian method implemented in BayeScan v.2.1 (Foll & Gaggiotti, 2008) that estimates the probability that a given locus is under selection based on a set of neutral allele frequencies approximated by a Dirichlet distribution. BayeScan was run five times with a burn-in of 50,000, a thinning interval of 10, and a sample size of 5000 (default parameters). The prior odds of the neutral model were raised to 1000 and the false discovery rate (FDR) was lowered to 1% to limit high false positives occurring with this method if populations underwent a bottleneck during a geographic expansion, which may be the case for the North American populations in our dataset (Foll & Gaggiotti, 2008). Only SNPs detected as outliers in the five different runs were kept as putatively selected SNPs identified by BayeScan. Then, we ran the software OutFLANK (Whitlock & Lotterhos, 2015), which identifies *F*_st_ outliers by inferring a distribution of neutral *F*_st_’s using likelihood on a trimmed distribution of *F*_st_ values. In comparison with BayeScan, OutFLANK displays lower false positive rates when populations experience range expansion (Lotterhos & Whitlock, 2014). The software was run with default parameters, except for the left-trimmed fraction of the *F*_st_ distribution, which was set to a higher value (LeftTrimFraction = 0.25), and a q-value ≤ 0.05 for a locus to be considered an outlier. Finely, we used the R package *pcadapt* (Luu et al., 2017), which first assesses population structure using principal component analysis (PCA) and then identifies markers that are excessively correlated with population structure as those under putative divergent selection. The *K* number of principal components necessary to appropriately describe the population structure was defined using Cattell’s rule as suggested by Luu et al. (2017). A SNP was considered an outlier when its q-value was < 0.1. By contrast with population-based approaches, *pcadapt* does not require that individuals be grouped into discrete populations and handles admixed individuals, a particularly useful feature in the current study as we expected some hybrid zones Luu et al. (2017). SNPs identified as divergent by at least two methods were included in the outlier set.

*Results*

BayeScan detected 808 putative outlier SNPs, representing 34.6 % of the SNP data set. Given this very high positive rate, calculations were redone after removal of North American samples from the dataset. Indeed, US and Canadian populations are suspected of having been subjected to a strong bottleneck effect during the geographic expansion that followed the accidental introduction that gave rise to them; such circumstances are known to result in high false-positive rates in BayeScan (Foll & Gaggiotti, 2008). Following this operation, the number of putative outlier SNPs decreased to 473 (20.26%), including 340 SNPs (14.56%) putatively under divergent selection and 133 SNPs (5.7%) putatively under balancing selection. The second method implemented in OutFLANK failed to detect any outlier SNP with a q-value ≤ 0.05; this situation is known to occur when neutral markers are strongly differentiated (see results in main document), i.e. when the right tail of the neutral *F*_st_ distribution is relatively long and broad (right-skewed distribution; Fig 1A left panel following page), making it difficult to statistically detect high *F*_st_ outlier SNPs (Whitlock & Lotterhos, 2015). Using a less conservative criterion to establish a threshold (*p*-value ≤ 0.05), OutFLANK identified 25 putatively divergent SNPs. Lastly, eight principal components (PCs) were retained to explain population structure in *pcadapt*, while 128 SNPs were significantly correlated with these eight PCs, resulting in their identification as putatively divergent SNPs. The number of outliers detected by each method and the overlap between the different methods are illustrated in Fig. 1 B, using the R function *venn.diagram* (R package Venn Diagram; (Chen & Boutros, 2011). At the end of this process, the neutral and divergent sets were considered to consist of 2,125 and 77 SNPs, respectively.

**References**

Chen, H., & Boutros, P. C. (2011). VennDiagram : A package for the generation of highly-customizable Venn and Euler diagrams in R. *BMC Bioinformatics*, *12*(1), 35. DOI:10.1186/1471-2105-12-35

Foll, M., & Gaggiotti, O. (2008). A genome-scan method to identify selected loci appropriate for both dominant and codominant markers : a bayesian perspective. *Genetics*, 180(2), 977‑993. DOI: 10.1534/genetics.108.092221

Lotterhos, K. E., & Whitlock, M. C. (2014). Evaluation of demographic history and neutral parameterization on the performance of FST outlier tests. *Molecular Ecology*, 23(9), 2178‑2192. DOI: 10.1111/mec.12725

Luu, K., Bazin, E., & Blum, M. G. B. (2017). pcadapt : An R package to perform genome scans for selection based on principal component analysis. *Molecular Ecology Resources*, 17(1), 67‑77. DOI: 10.1111/1755-0998.12592

Whitlock, M. C., & Lotterhos, K. E. (2015). Reliable Detection of Loci Responsible for Local Adaptation : inference of a null model through trimming the distribution of FST. *The American Naturalist*, 186(S1), S24‑S36. DOI: 10.1086/682949

(A)
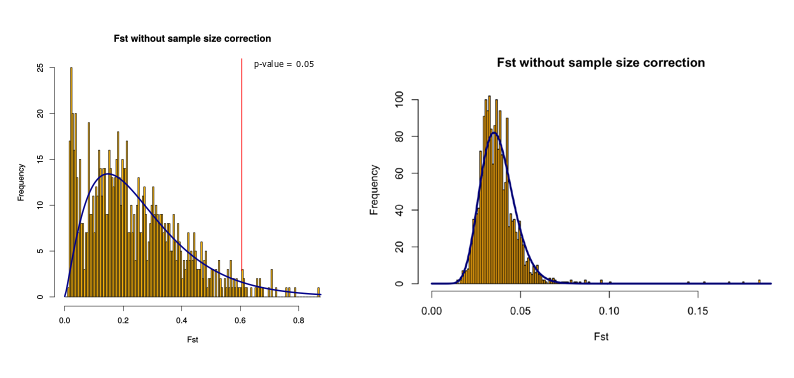


Present study data set “Ideal” data set

(B)

77 SNPs


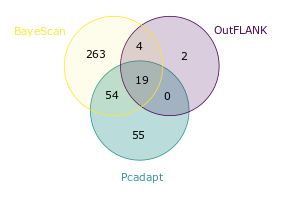


**Figure 1.** **Detection of loci under putative selection.** (A) Results of OutFLANK analysis. The bar chart shown on the left is an *F*_st_ histogram without sample correction of loci, with a minimum expected heterozygosity He > 0.1. The blue curve is the inferred distribution of *F*_st_ for neutral markers calculated on the trimmed distribution of *F*_st_ values. The histogram shows a relatively long and broad right tail of the neutral *F*_st_ distribution with many high *F*_st_ SNPs (> 0.3). This kind of *F*_st_ distribution results in a right-skewed curve for the inferred distribution of *F*_st_, making it difficult to statistically detect high *F*_st_ outlier SNPs (Whitlock & Lotterhos, 2015) with a conservative threshold, i.e. q-value ≤ 0.05 (no outlier detected). With a less conservative threshold, i.e. p-value ≤ 0.05 (vertical red line), we were able to identify 25 putative divergent SNPs. The histogram shown on the right is an example of an *F*_st_ distribution where OutFLANK typically performs well (Lotterhos E., [Tutorial_OutFLANK](http://rstudio-pubs-static.s3.amazonaws.com/305384_9aee1c1046394fb9bd8e449453d72847.html), last accessed 09/09/2020). **(**B) Number of SNPs identified as putatively under divergent selection using three genome scan methods. The SNPs included in the divergent SNP set are those identified by at least two methods (54 + 19 + 4 = 77 SNPs).

**Supplementary information 2: minimum sample size for computation of accurate gene diversity (*unbiased D*) estimates.**

A subsampling technique was used to investigate the effect of sample size (i.e. number of moths considered) on estimates of unbiased gene diversity (*D* or expected heterozygosity *He*; Nei & Roychoudhury, 1974) calculated on the neutral dataset (2125 SNPs). Specifically, we selected 10 populations featuring a minimum sample size of 28 moths, and considered representative of the diversity of our sampling effort (AT02, CA04, CN01, CN05, EE01, FR06, PL01, SK01, SP01, and TR01). For each population, we randomly sampled without replacement n individuals (*n* = 4, 6, 8, 10, 15, 20, and 25). Then, we estimated gene diversity as calculated on *n* individuals. We repeated this operation 100 times for each value of *n*. The gene diversity estimates were calculated with the function *Hs()* of the R package *adegenet* (Jombart & Ahmed, 2011) and were multiplied by 2n/(2n-1) to obtain unbiased estimates. Notched box plots were used to assess the effects of sample size on gene diversity estimates. The notch of a box plot displays a 95% confidence interval around the median (median ± IQR/√n; IQR = interquartile range i.e. 25 to 75 percentile); if two boxes’ notches do not overlap, the medians are considered different (Chambers, 1983). Gene diversity estimates calculated for 25 individuals were not significantly different from those calculated for 15 or 20 individuals, based on the populations included in this analysis, with the exception of TR01 for which 4 individuals were sufficient (see Fig. 1 below). Therefore, for computation of gene diversity estimates, we included only populations with ≥ 15 individuals, which represents a compromise between high population sample size and reliable gene diversity estimates.

**Figure 1
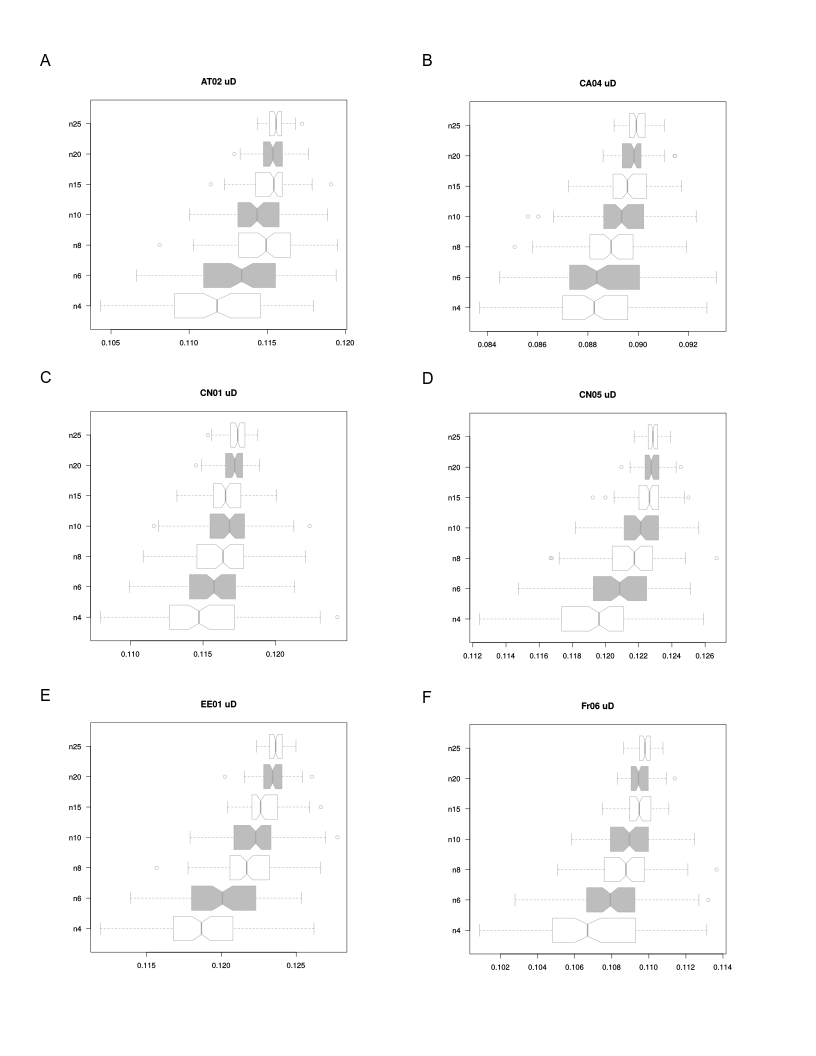
**

**Figure 1 continued**

**
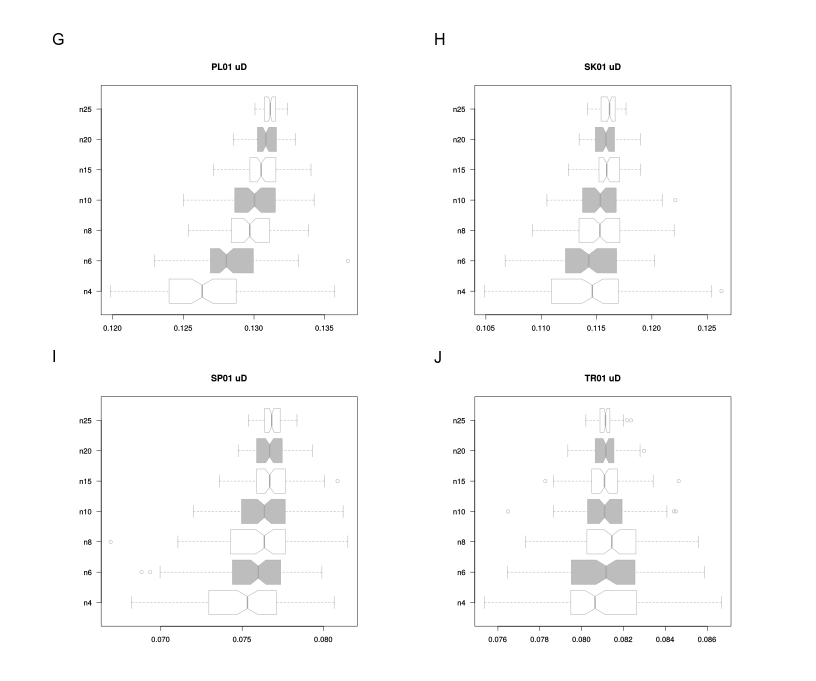
**

**Figure 1.** Distribution of differences in unbiased gene diversity (*unbiased D*) estimates calculated for samples sizes n = 4, 6, 8, 10, 15, 20 and 25, in 10 different populations (A - J).

**References**

Chambers, J. M., Cleveland, W. S., Kleiner, B., & Tukey, P. A. (1983). *Graphical Methods for Data Analysis*. Wadsworth International Group..

Jombart, T., & Ahmed, I. (2011). adegenet 1.3-1 : New tools for the analysis of genome-wide SNP data. *Bioinformatics (Oxford, England)*, *27*(21), 3070‑3071. 345. https://doi.org/10.1093/bioinformatics/btr521

Nei, M., & Roychoudhury, A. K. (1974). Sampling variance of heterozygosity and genetic distance. *Genetics*, *76*(2), 379. 346.

**Supplementary information 3: Admixture analysis and Kmeans method: K=9-10 and outlier dataset**

Based on neutral SNPs, the *k-mean* methods implemented in the *find.clusters* indicated that the most likely number of genetically distinct clusters, was *K* = 8 or 9 (lowest BIC median and lowest BIC values, respectively) (Fig. S5) whereas the maximum-likelihood approach implemented in ADMIXTURE v1.3.0, the minimum number of groups varied between *K* = 8-10 once corrected for spurious group (Guillot et al., 2005; Puechmaille, 2016). For the *K*_corrected_ = 9-10 obtained with ADMIXTURE, the most frequently observed subdivisions were: (i) two clusters in North America, (ii) the Estonian population forming a distinct group, (iii) western and central European populations split into two subgroups (France, Belgium and west Germany *vs* Italy, Slovenia, Austria, Slovakia, Kosovo and Bulgaria), and (iv) the east Asian cluster further subdivided into two groups (from south-east to north-east China *vs* far north-east China (CN04), the Russian Far East and Korea). For *K* = 9 with the *k-*mean methods, the same East Asian subdivision is also identified by the three runs yielding lower BIC values than the median of the BIC values at *K* = 8.

Using the outlier SNP set, ADMIXTURE identified *K* = 7 as the most likely number of groups, while with the *k-mean* method, the BIC value curve flattened at values of *K* = 15-17 (Fig. S4). The groups identified by both methods agree with those found with the neutral dataset. The additional groups identified by the *k*-mean method were mainly found in Europe and western Russia. It must be noted that the boundaries between these additional groups were blurred, i.e. there were always a few individuals from a cluster that grouped with the closest neighboring clusters (Fig. S4).

**References**

Guillot, G., Estoup, A., Mortier, F., & Cosson, J. F. (2005). A Spatial Statistical Model for Landscape Genetics. *Genetics*, *170*(3), 1261. 341. https://doi.org/10.1534/genetics.104.033803

Puechmaille, S. J. (2016). The program structure does not reliably recover the correct population structure when sampling is uneven : Subsampling and new estimators alleviate the problem. *Molecular Ecology Resources*, *16*(3), 608‑627. 101. https://doi.org/10.1111/1755-0998.12512

**Supplementary information 4: TreeMix – alternative tree and detailed description of the migration events**

1. Example of a TreeMix tree rooted with a different East Asian spongy moth population (RU39).


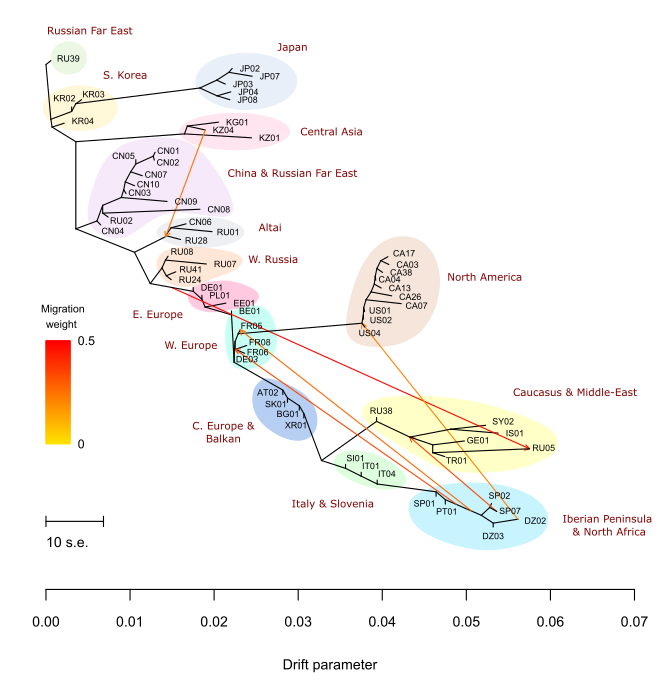


Maximum likelihood population graph tree generated by TreeMix rooted with the population showing the highest gene diversity value (RU39), inasmuch as high genetic diversity is expected for populations found in a species’ geographic origin.

1. Detailed description of migration events.

Regarding the migration events identified by Treemix, the analysis seems to confirm cases of admixture also identified by ADMIXTURE analysis in populations at the edges of the different population clusters (see main text). More precisely, genetic contributions from southern Spain and Algeria to French populations were inferred (FR05: w = 26%, p-value = 6.99 x 10^-15^; FR06/FR08: w = 34%, p-value ≤ 10^-30^) whereas a southern Spain population (SP07) contributed to Caucasian and Middle Eastern populations (w = 34%, p-value ≤ 10^-30^). In addition, a Kazakhstan population (KZ04) genetically contributed to a Siberian population (RU28: w = 24%, p-value = 3.44 x 10^-12^), and an important migration event was also identified from European populations to a Russian population in the Caucasus (RU05: w = 48%, p-value ≤ 10^-30^). For the latter migration event, the RU05 population consisted in only two individuals and in the ADMIXTURE and PCA analyses, one of these individuals was indeed identified as genetically closest to Eastern Europe (Poland and West Germany) populations, explaining the high weight of this migration event as determined by TreeMix analysis.

**Supplementary information 5: Fine population structure explored with DAPC and Assignment analysis**

Based on neutral SNPs, the maximum-likelihood approach implemented in ADMIXTURE v1.3.0 and the *k-mean* methods implemented in the *find.clusters* identified the same minimum 8 groups: (1) North-America, (2) Iberian peninsula and Algeria, (3) West and central Europe (France, Belgium, West Germany, Italia, Slovenia, Austria, Slovakia, Kosovo, Bulgaria), (4) Caucasus and Middle East, (5) northern Europe (north-east Germany, Poland, Estonia) and western Russia up to Western Central Asia (Novosibirsk region in Russia), (6) Eastern Central Asia (Kazakhstan, Kyrgyzstan, Altay region in Russia and China), (7) continental east Asia and (8) Japan. For each of these 8 groups, lower-level structures were explored using Discriminant Analysis of Principal Components (DAPC; Jombart et al., 2010); the pertinent number of principal components retained for these analyses was submitted to a cross-validation test using the R function *xvalDapc* (R package *adegenet*; Jombart et al., 2010). To assess the significance of the sub-groups identified by DAPC, assignment analyses were carried out using the R function *predict.dapc* (R package *adegenet*; Jombart et al., 2010), which is based on the outcome of DAPC analysis. To conduct assignment analysis, we used a 10-fold cross-validation procedure to limit overestimation of membership probability. This cross-validation divided the dataset in 10 groups and, then, one of the 10 groups was tested by the *predict.dapc* function based on the DAPC results carried out on the remaining 10-1 groups. These assignment tests were repeated until every group was tested. We considered that a sub-group (one or several populations) was sustained when 80 % of its individuals attained a posterior probability of membership ≥ 0.8. For each region, the DAPC scatter plot (A) shows the individuals as dots and the populations as inertia ellipses; eigenvalues are shown in the inset. Assignment results (B) are presented in the form of a contingency table where columns correspond to the original populations, while rows correspond to inferred populations. The position of each black square indicates the population to which individuals were assigned (population showing the highest posterior probability of membership) while the size of each square is proportional to the number of individuals so assigned. The red dotted line delimits the sustained subgroups according to the criteria referred to above. The values in red characters represent the percentage of individuals attaining a posterior probability of membership ≥ 0.8. In continental East Asia and Japan, the percentage of correct assignment of two groups increased sharply when we used the highest posterior probability of membership as criterion for correct assignment instead of the 0.8 threshold. We identified the groups in question and the new percentage of correct assignment in blue characters.

1 – *North America*: 2 subgroups


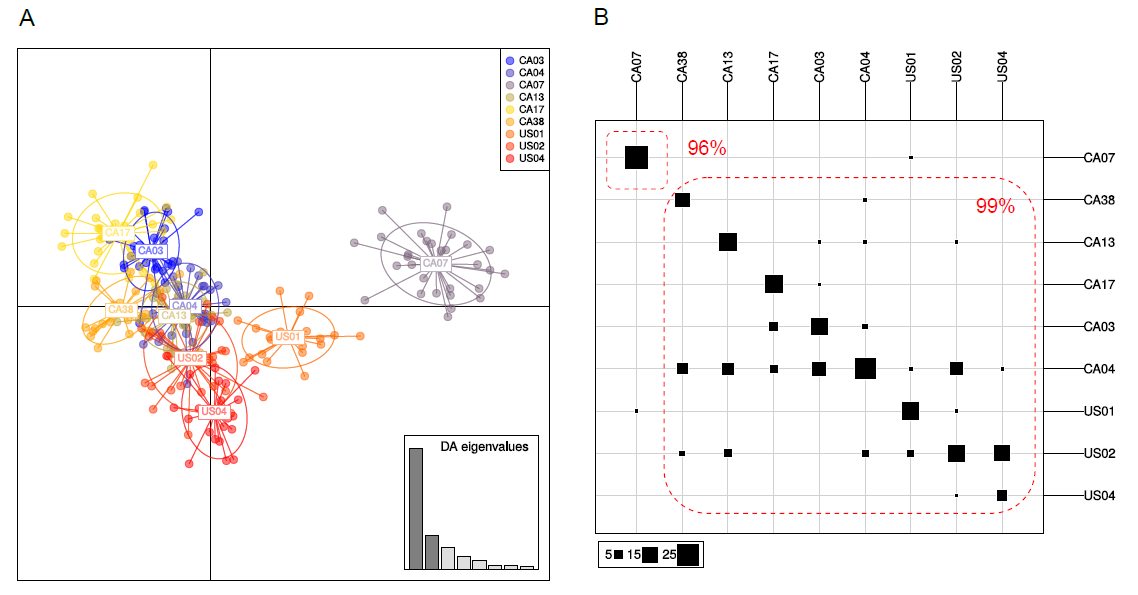


2 - *Iberian peninsula and Algeria*: 5 subgroups


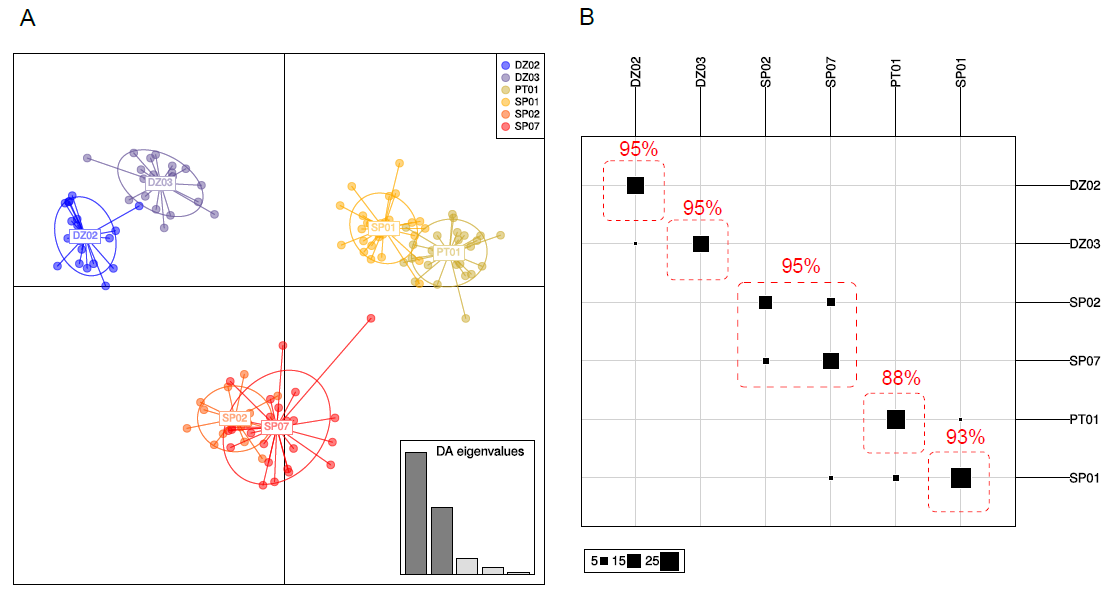


3 - *West and central Europe*: 4 subgroups


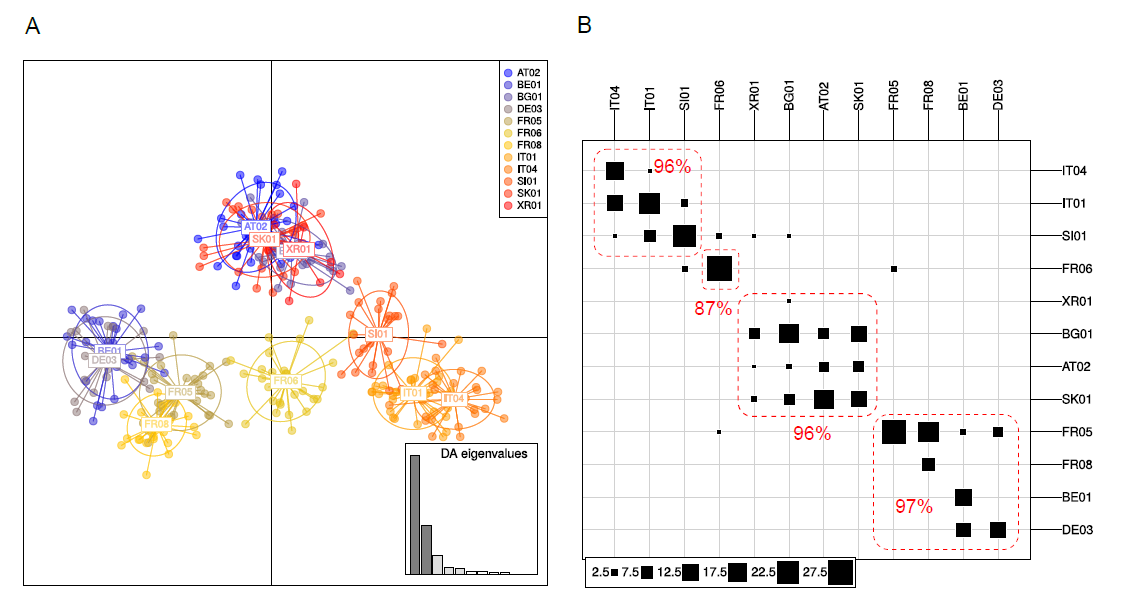


4 - *Caucasus and Middle East* – 4 subgroups *
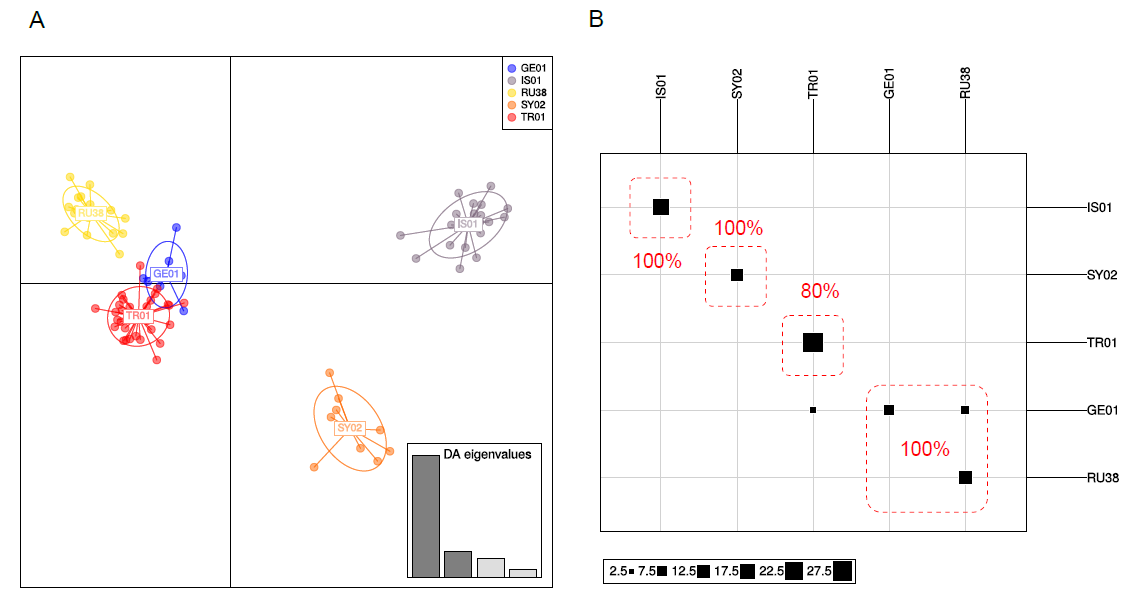
*

5 - *Northern Europe* *and western Russia* – 3 subgroups


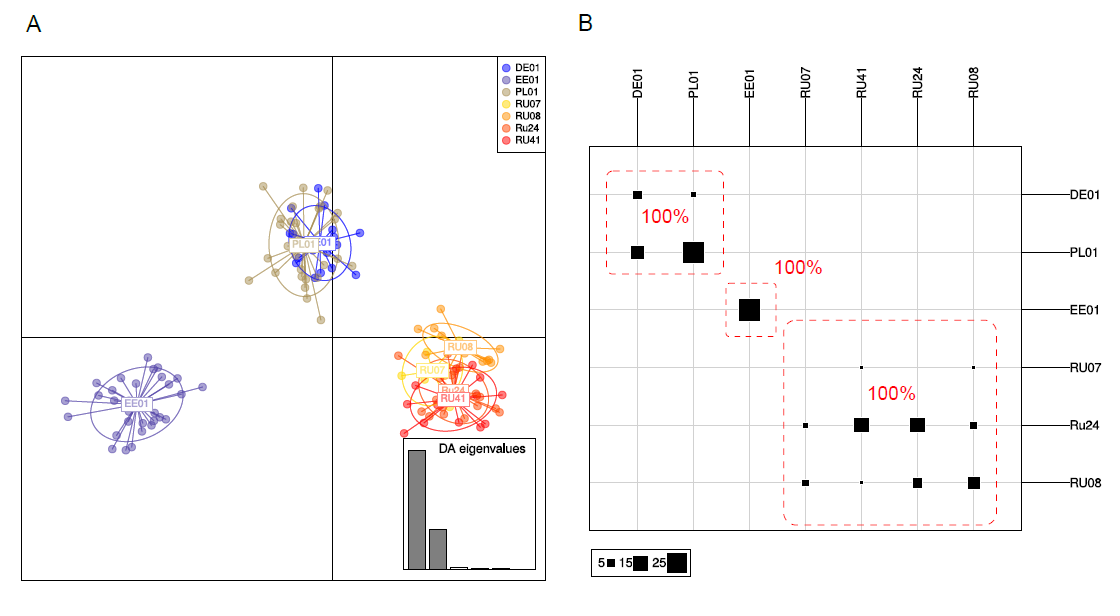


6 - *Eastern Central Asia* – 3 subgroups


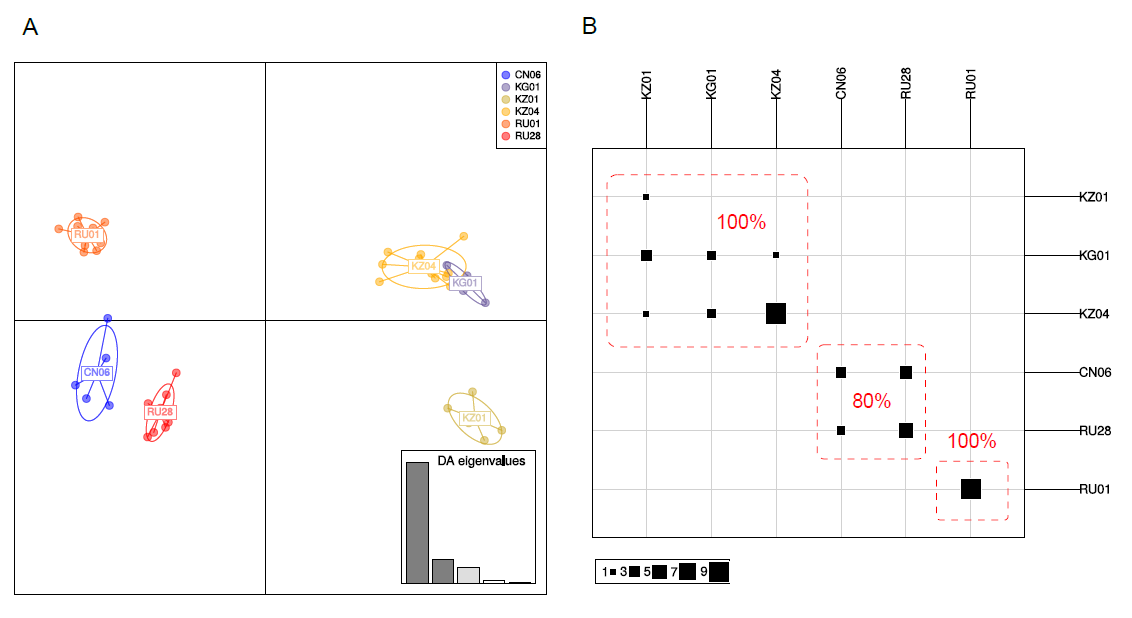


8 - *Japan* – 2 subgroups


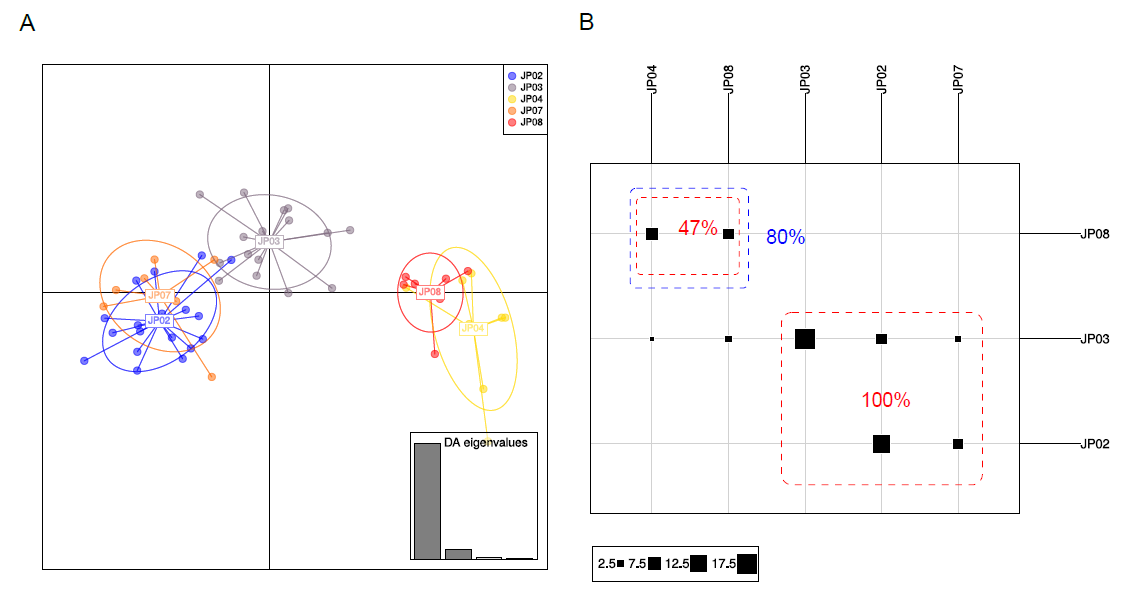


7 – *Continental East Asia* – 5 subgroups


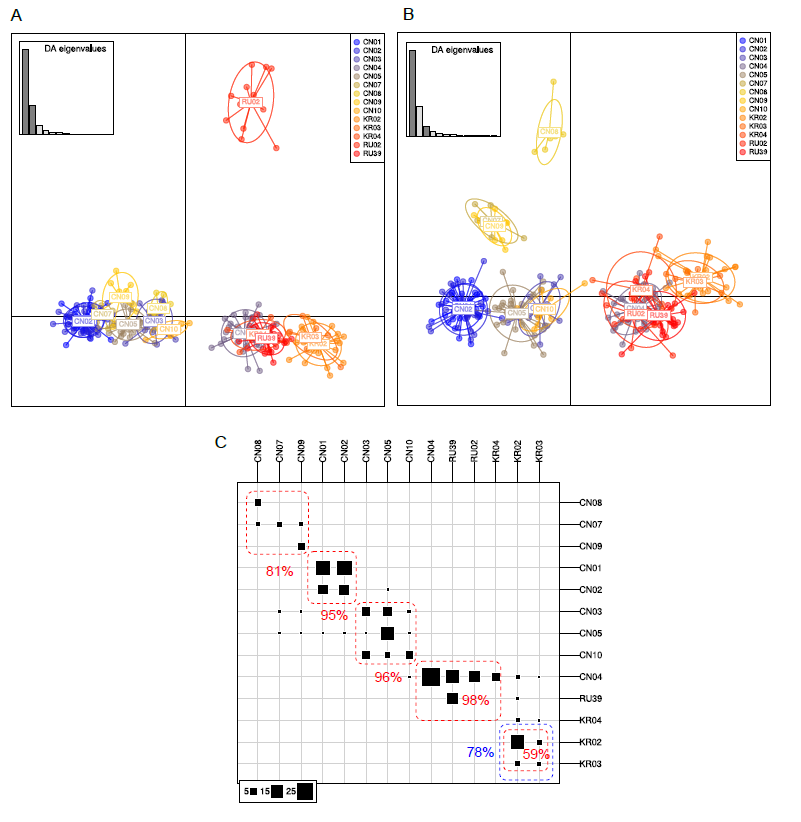

Supplement: Supplementary file 1 — Appendix S1 [file EVA-16-638-s001.docx]
